# Molecular Lineages and Spatial Distributions of Subplate Neurons in the Human Fetal Cerebral Cortex

**DOI:** 10.1002/advs.202407137

**Published:** 2024-11-04

**Authors:** Xueyu Guo, Trevor Lee, Jason Sun, Julianne Sun, Wenjie Cai, Qingwei Yang, Tao Sun

**Affiliations:** ^1^ Center for Precision Medicine Huaqiao University Xiamen Fujian 361021 China; ^2^ Department of Cell and Developmental Biology Weill Medical College of Cornell University 1300 York Avenue New York NY 10065 USA; ^3^ Xiamen Institute of Technology Attached School Xiamen Fujian 361005 China; ^4^ Department of Radiation Oncology First Hospital of Quanzhou Fujian Medical University Quanzhou Fujian 362046 China; ^5^ Department of Neurology Zhongshan Hospital School of Medicine Xiamen University Xiamen Fujian 361006 China; ^6^ School of Medicine and School of Biomedical Sciences Huaqiao University Xiamen Fujian 361021 China

**Keywords:** cerebral cortex, human fetal brain, single‐cell RNA‐sequencing, spatial transcriptomics, subplate neurons

## Abstract

The expansion of neural progenitors and production of distinct neurons are crucial for architectural assembly and formation of connectivity in human brains. Subplate neurons (SPNs) are among the firstborn neurons in the human fetal cerebral cortex, and play a critical role in establishing intra‐ and extracortical connections. However, little is known about SPN origin and developmental lineages. In this study, spatial landscapes and molecular trajectories of SPNs in the human fetal cortices from gestational weeks (GW) 10 to 25 are created by performing spatial transcriptomics and single‐cell RNA sequencing. Genes known to be evolutionarily human‐specific and genes associated with extracellular matrices (ECMs) are found to maintain stable proportions of subplate neurons among other neuronal types. Enriched ECM gene expression in SPNs varies in distinct cortical regions, with the highest level in the frontal lobe of human fetal brains. This study reveals molecular origin and lineage specification of subplate neurons in the human fetal cerebral cortices, and highlights underpinnings of SPNs to cortical neurogenesis and early structural folding.

## Graphical abstract

This study applies spatial transcriptomics and single‐cell RNA sequencing to study molecular heterogeneity of subplate neurons (SPNs) in human fetal brains from gestational week 10 to 25. Human‐specific and extracellular matrices (ECM) genes are found to maintain SPN population. Enriched ECM gene expressions in the frontal lobe suggest a potential role of SPNs on regional cortical folding.

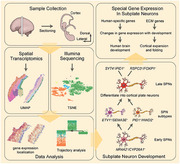

## 1 Introduction

The human brain is a complex convoluted structure consisting of various neuronal and glial cell types.^[^ ^1^, ^2^, ^3^ ^]^ Precise regulations of expansion of radial glial cells (RGCs) and intermedial progenitor cells (IPCs), and subsequent production and migration of distinct neurons in the human fetal cerebral cortex ensure assembly of multiple layered cortical architecture, and establishment of sophisticated cognitive functions.^[^ ^1^, ^2^, ^4^, ^5^, ^6^ ^]^ An arsenal of tools such as morphology, electrophysiology, imaging, particularly single‐cell RNA sequencing (RNA‐seq) and spatial transcriptomics have created rich and visible landscapes of cell origin and lineages in the human developing brain.^[^ ^7^, ^8^, ^9^ ^]^

Within the human fetal cortical laminae, the subplate is a morphological definition based on cytoarchitecture.^[^ ^10^, ^11^ ^]^ The subplate, which consists of subplate neurons (SPNs), is a transient layer between the emergent cortical plate and underlying white matter.^[^ ^11^, ^12^, ^13^, ^14^ ^]^ The origin of human SPNs is still not certain, even though it is known to be prominent in fetal cortices from 17 to 37 gestational weeks (GW).^[^ ^11^, ^15^, ^16^, ^17^ ^]^ In humans, the subplate appears to be split from a superficial compartment of the intermediate zone termed the presubplate, which is considered the first postmitotic cortical neuronal layer, at GW9‐10.^[^ ^4^, ^18^, ^19^, ^20^ ^]^ Because the subplate is a highly dynamic sector of the developing cortex, gene expression profiles have identified very few specific markers for SPNs, which indicates molecular heterogeneity and evolutionary complicity of SPNs in different species.^[^ ^21^, ^22^, ^23^, ^24^, ^25^ ^]^ Subplate neurons play a critical role in the establishment of the very first neural circuit formation between the thalamus and cortex.^[^ ^12^, ^26^ ^]^ During cortical development, SPNs receive thalamic and intra‐cortical input, and primarily project to Layer 4 (L4).^[^ ^11^, ^19^, ^27^, ^28^, ^29^ ^]^ Studies have shown that SPNs likely play an instructive role in thalamocortical and intracortical connectivity, and these neural activities in turn regulate functional maturation of neurons and neural circuits.^[^ ^19^, ^27^, ^30^, ^31^ ^]^

Moreover, the subplate is abundant with extracellular matrices (ECMs), making it a signaling center for the formation of connectivity in the cerebral cortex.^[^ ^20^, ^32^, ^33^ ^]^ As ECM components are reported to regulate expansion of cortical neural progenitors and migration of neurons, the subplate is proposed to have a role in facilitating folding of the human fetal cerebral cortex.^[^ ^34^, ^35^, ^36^ ^]^ Noticeably, proper connectivity to SPNs may have a significant impact to synaptic plasticity in the human brain, and is likely associated with neuropsychiatric disorders, for instance, autism and schizophrenia.^[^ ^13^, ^20^, ^37^, ^38^ ^]^ However, the origin and molecular trajectories of subplate neurons in the human fetal brain remain obscure.

To reveal molecular landscapes of human subplate neurons, we performed spatial transcriptomics and single‐cell RNA‐seq in human fetal cerebral cortices from GW10 to GW25, and generated spatial and molecular lineage profiles of cortical SPNs. We discovered stable and continuous expressions of both human‐specific genes and ECM genes in SPNs from GW14 to GW20, and detected enriched ECM gene expressions in SPNs in the frontal lobe, suggesting a potential role of SPNs on regional expansion of the subplate and cortical folding during human fetal brain development. Our results of spatial transcriptomics and single‐cell RNA‐seq have revealed precise positional information of cortical subplate neurons as well as their high diversity and trajectories in the human fetal cerebral cortex.

## 2 Results

### 2.1 Spatial Distributions of Marker Genes in the Human Fetal Cortex

**Figure 1:**
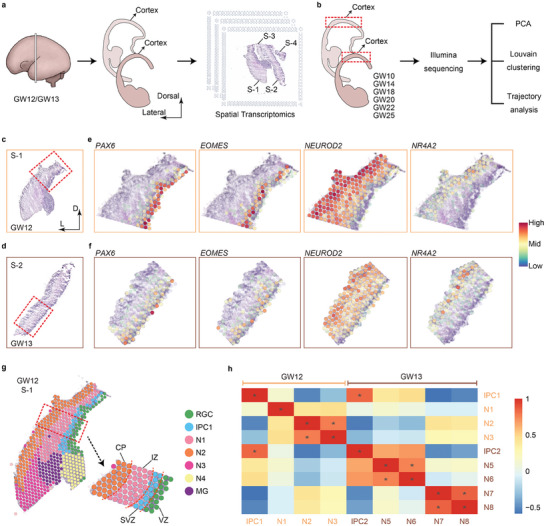
Spatial distributions of various cell types in the human fetal cortices detected by spatial transcriptomics. a) Workflow of spatial transcriptomic assays in the human fetal cerebral cortices at GW12 and GW13. b) Analysis processes of single‐cell RNA‐seq datasets from human fetal cerebral cortices collected from GW10 to GW25 (8 stages). c, d) Two cortical sections: S‐1 at GW12 and S‐2 at GW13. The boxed areas in c and d are displayed in high‐power views in e and f. e, f) Spatial spots of expression patterns of *PAX6*, *EOMES*, *NEUROD2*, and *NR4A2* in the S‐1 and S‐2 sections in human feal cortices at GW12 and GW13, respectively. Each spot represents signals of gene expression in the cortical wall. g) Cell clusters and cell spatial distributions in the cortex based on spatial spots annotated in the S‐1 section at GW12, with radial glial cells (RGCs) in the ventricular zone (VZ), intermediate progenitor cells (IPC1) in the subventricular zone (SVZ), and neurons (N1‐N4) in the intermediate zone (IZ) and cortical plate (CP). h) Pearson correlation coefficient analysis between intermediate progenitor cells (IPCs) and neurons (N) from S‐1 to S‐4 sections in human fetal cortices at GW12 and GW13. Correlation coefficient ≥ 0.7, *P* < 0.05.

To uncover the developmental process of subplate neurons (SPNs) in the human cerebral cortex at its early fetal stages, we performed spatial transcriptomics and single‐cell RNA sequencing (RNA‐seq) in human cortices from gestational weeks (GW) 10 to 25 (GW10, GW12, GW13, GW14, GW18, GW20, GW22, and GW25) (**Figure** **1**a,b).^[^ ^39^ ^]^ Compared to post‐conceptional weeks (PCWs), gestational weeks are two weeks earlier than PCWs. We prepared brain sections at GW12 and GW13, and applied 10x Genomics spatial transcriptomic technology to capture gene expression patterns as spatial spots in situ (Figure 1a). We analyzed 4 brain sections, including section‐1 (S‐1) from GW12, and section‐2 to section‐4 (S‐2, S‐3 and S‐4) from GW13, and constructed spatial gene expression images by plotting 1162 spatial spots.

We selected representative S‐1 and S‐2 sections at human fetal GW12 and GW13, respectively, and applied graph‐based Louvain clustering to spatial spots. 12 cell clusters such as radial glial cells (RGCs), intermediate progenitor cells (IPC1 and IPC2), neurons (N1‐N8), and microglia (MG) were annotated according to known cortical neuronal markers (Figure S1a and Table S1, Supporting Information).^[^ ^4^, ^40^, ^41^ ^]^ RGCs were identified in both sections, and cells for instance IPC1, N1‐N4, and MG were highly detected in the S‐1 section, and cells of IPC2 and N5‐N8 were observed in the S‐2 section (Figure S1a, Supporting Information). To examine expression patterns of known genes in various cortical cell types, we looked into spatial spots of *PAX6*, *EOMES* (also known as *TBR2*), and *NEUROD2* in both S‐1 and S‐2 sections (Figure 1c–f). Spatial spots of *PAX6* in RGCs were observed in the ventricular zone (VZ), and spatial spots of *EOMES* in IPCs and *NEUROD2* in neurons were detected in the subventricular zone (SVZ) and in the intermediate zone (IZ) and cortical plate (CP), respectively, in both sections (Figure 1e,f). Interestingly, spatial spots of *NR4A2* (also known as *NURR1*), a representative marker in subplate neurons,^[^ ^22^ ^]^ was mainly detected in the cortical plate (Figure 1e,f). Similar spatial expression patterns of *PAX6*, *EOMES*, *NEUROD2*, and *NR4A2* were observed in the S‐3 and S‐4 sections, compared to those in the S‐1 and S‐2 (Figure S1b,c, Supporting Information). These results suggest that spatial transcriptomics can reveal precise distributions of genes expressed in distinct cell types in the human fetal cortex at different stages, in particular RGCs in the VZ, and subplate neurons in the CP (Figure 1g; Figure S1d, Supporting Information).

To obtain an intuitive understanding of the cell clusters captured by spatial transcriptomics, we merged all spatial spots from four sections, visualized them with Uniform Manifold Approximation and Projection (UMAP), and identified expression patterns of *PAX6*, *EOMES*, *NEUROD2*, and *NR4A2* in each cell cluster (Figure S2a–c, Supporting Information). Notably, SPN markers such as *ST18* and *ZFPM2* were expressed in the cluster of *NR4A2*,^[^ ^22^, ^42^ ^]^ further indicating presence of subplate neurons (Figure S2c, Supporting Information).

Next, to reveal the correlation between intermediate progenitor cells (IPC1 and IPC2) and neurons (N1‐N3 and N5‐N8) in cortices from GW12 and GW13, we screened and compared 690 differentially expressed genes (DEGs) (log_2_ fold change > 0.25, pct.1 > 0.6 and pct.2 < 0.4) (Tables S2 and S3, Supporting Information). We obtained a correlation coefficient matrix between each cell cluster, and marked the cell clusters with strong correlation with asterisks (coefficient ≥ 0.7, *P* < 0.05) using the Pearson correlation coefficient calculation function (Figure 1h; Table S4, Supporting Information). Even though IPC1 and IPC2 are from cortices at GW12 and GW13, respectively, they displayed a strong correlation, suggesting a continuous expansion of cortical intermediate progenitors at different developmental stages (Figure 1h). Moreover, cortical neurons (N1‐N8) showed a significant heterogeneity, in particular between the N2 and N3 populations, indicating dynamic neurogenesis stages from GW12 to GW13 (Figure 1h).

In summary, our results of spatial transcriptomics reveal accurate positional information of various cortical cell types and their high diversity in the human fetal cerebral cortex.

### 2.2 Molecular Characteristics of Subplate Neurons

**Figure 2:**
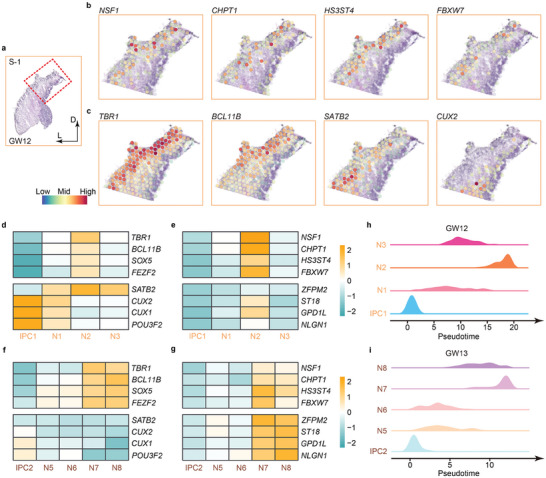
Spatial characteristics of subplate neurons in human fetal cortices at GW12 and GW13. a) The S‐1 cortical section at GW12. The boxed area in a is displayed in high‐power views in b and c. b) Spatial spots of expression patterns of representative SPN genes in the cortical plate in the GW12 cortex. c) Spatial spots of expression patterns of markers for early‐born and late‐born neurons in the cortical plate in the GW12 cortex. d‐g) Heatmaps of expression levels of markers for early‐born neurons, late‐born neurons, and subplate neurons in cell clusters annotated from genes expressed in the GW12 (d, e) and GW13 (f, g). h,i) Developmental pseudotime ridge plots of intermediate progenitor cells (IPCs) and neurons (N) captured from the S‐1 cortical section at GW12 (h) and those from S‐2, S‐3, and S‐4 cortical sections at GW13 (i).

To uncover the spatial distribution of subplate neurons in the human fetal cerebral cortex, we searched previously reported 124 SPN marker genes, and detected 8 of them highly expressed at GW12, and 30 of them at both GW12 and GW13, as captured by spatial transcriptomics (pct.1 > 0.6 and pct.2 < 0.4) (Tables S5–S7, Supporting Information).^[^ ^22^ ^]^ We selected 4 representative genes *NSF1*, *CHPT1*, *HS3ST4*, and *FBXW7* with high expression at GW12 and 12 representative genes at both GW12 and GW13, and examined their spatial distributions in the S‐1 and S‐2 sections (Tables S6 and S7, Supporting Information). We observed their spatial positions in the cortical plate in the S‐1 section at GW12 and S‐2 at GW13, suggesting that subplate neurons are mainly positioned in the CP in early human fetal cortices (**Figure** **2**a,b; Figure S3, Supporting Information).

We then compared spatial information of SPNs with early‐born neurons expressing *TBR1* and *BCL11B* (also known as *CTIP2*), and late‐born neurons expressing *SATB2* and *CUX2*.^[^ ^41^, ^43^ ^]^ *TBR1*, *BCL11B*, and *SATB2* were highly expressed in the CP with scattered expression in the IZ, while *CUX2* was mostly expressed in the SVZ and IZ in the S‐1 section at GW12, as detected by spatial transcriptomics (Figure 2a,c). Moreover, while the early‐born neurons expressing *TBR1* and *FEZF2* were concentrated in the N2 cluster in the S‐1 at GW12, the late‐born neurons expressing *CUX1* and *POU3F2* were classified in the IPC1 and N1 clusters, the subplate neurons expressing *NSF1*, *CHPT1*, and *ST18* were grouped in the N2 cluster (Figure 2d,e). Similarly, subplate neurons also were concentrated in N7 and N8 cell clusters in S‐2 to S‐4 sections at GW13, which also were enriched by early‐born neurons (Figure 2f,g; Table S8, Supporting Information). These results suggest that subplate neurons display spatial and cell cluster features more closely aligned with those of early‐born neurons in the human fetal cortices at GW12 and GW13.

To further investigate a temporal relationship among early‐born, late‐born neurons and subplate neurons, we visualized pseudotime developmental trajectories of intermediate progenitor cells and neurons in S‐1 to S‐4 sections at GW12 and GW13 using ridge plots. We observed that IPC1 is located at the starting point of the trajectory, with a sequential generation of N1, N2, and N3 neurons, and IPC2 at the starting point of the trajectory with a sequential differentiation of N5/N6, towards N8 and N7, in the human fetal cortices at GW12 and GW13, respectively (Figure 2h,i). Because late‐born neurons were more clustered in the IPCs, and subplate neurons were enriched in the N2 and N7/8 clusters, these results indicate an early‐born neuron‐like population of SPNs in the human fetal cortices, suggesting the presubplate stage of cortical development at GW12 and GW13.^[^ ^4^, ^18^, ^19^ ^]^ Our results further supported previous studies, in which cells expressing subplate markers were situated near or within the CP at stages GW12‐15.^[^ ^18^, ^20^, ^25^, ^44^ ^]^

In summary, spatial transcriptomics has generated a spatial and temporal atlas of early‐born, late‐born neurons and subplate neurons in human fetal cortices, and has revealed a closer feature of subplate neurons to early‐born neurons than to late‐born neurons.

### 2.3 The Origin of Early Subplate Neurons

To further reveal molecular signatures of subplate neurons in distinct cortical regions at different fetal stages, we collected and integrated datasets of single‐cell RNA‐seq captured from human fetal cortices at GW10‐GW25 using principal component analysis (PCA) dimensionality reduction, Louvain clustering, and trajectory analysis (Figure 1b; Figure S4a, Supporting Information). Eight cortical regions including prefrontal cortex (PFC), motor, central, somatosensory, parietal, temporal, occipital, and primary visual (V1) cortex in the human fetal brains were analyzed using single‐cell RNA‐seq (Figure S4a, Supporting Information).^[^ ^39^ ^]^ 314 889 single cells were captured and 241 979 cells passed doublet detection and quality control (Table S9, Supporting Information).

**Figure 3:**
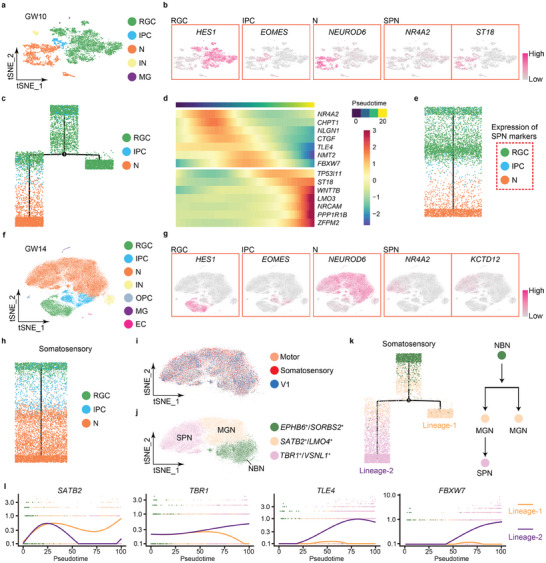
Origin of subplate neurons (SPNs) in the early human fetal cortices. a) Cell clusters of the human fetal cortex at GW10 based on single‐cell RNA‐seq datasets after Harmony integration depicted by t‐SNE. Each dot represents one cell. b) Known genes of RGCs, IPCs, neurons (N) and SPNs at GW10 visualized using t‐SNE. c) A lineage tree of RGCs, IPCs and neurons of single‐cell RNA‐seq datasets of the GW10 cortex. d) Pseudotime expression heatmap of 14 representative genes of subplate neurons from the GW10 cortex. e) A lineage tree of 14 SPN‐specific genes expressed in the RGCs, IPCs, and neurons. f) Cell clusters of the human fetal cortex at GW14 based on single‐cell RNA‐seq datasets. g) Known genes of RGCs, IPCs, neurons, and SPNs at GW14 visualized using t‐SNE. h) A lineage tree of RGCs, IPCs, and neurons of single‐cell RNA‐seq datasets from the somatosensory region of the GW14 cortex. i, j) Cell subclusters of neurons of single‐cell RNA‐seq datasets from the motor, somatosensory, and V1 regions of the GW14 cortex visualized by t‐SNE after batch correction. k) A lineage tree of newborn neurons (NBNs), migrating neurons (MGNs), and subplate neurons in the somatosensory region of the GW14 cortex. l) Changes in expression levels of *SATB2*, *TBR1*, *TLE4*, and *FBXW7* in the Lineage‐1 and Lineage‐2 along the pseudotime.

We first analyzed the datasets at GW10 using t‐distributed stochastic neighbor embedding (t‐SNE), and annotated five cell clusters as RGCs, IPCs, neurons (Ns), interneurons (INs), and MG, by expressing feature genes such as *HES1*, *EOMES*, *NEUROD2*, *DLX5*, and *SPP1*, respectively (**Figure** **3**a,b; Figure S4b, Supporting Information). To understand the developmental trajectory between RGCs, IPCs, and neurons, we constructed a lineage tree using Monocle 2, and found that RGCs are at the top of developmental trajectory, and are split into two branches, with one maintaining the RGC lineage and one sequentially producing RGCs, IPCs and then neurons (Figure 3c; Figure S4c, Supporting Information). Among these three types of cells in GW10 cortices, majority of cells were annotated as RGCs (64%), a small number of cells were neurons (31%), and the number of IPCs was the least (5%), indicating a cortical neural progenitor expanding stage at GW10 (Figure S4d, Supporting Information).

Interestingly, we didn't detect a cell cluster that is specific for subplate neurons. Instead, markers for SPNs were observed in clusters of both RGCs and neurons. For instance, *NR4A2* and *CYP26A1* were highly expressed in RGCs, and *ST18* and *SLC12A5* in neurons (Figure 3b; Figure S4e, Supporting Information). We examined the expression of 124 reported SPN markers, and found 113 of them detectable in the human fetal cortices at GW10.^[^ ^22^ ^]^ Among these 113 SPN genes, 110 genes were detected in RGCs, 86 in IPCs, and 111 in neurons (Table S10, Supporting Information). We then calculated the overall expression levels of 113 SPN markers in RGCs, IPCs, and in neurons, and identified 7 representative SPN markers with higher expression levels in RGCs and IPCs, and 7 with high expressions in neurons (Tables S11 and S12, Supporting Information). We mapped a pseudotime heatmap for these 14 SPN‐specific genes, performed trajectory analyses, and found that SPN genes expressed in RGCs such as *NR4A2* and *TLE4* are positioned at the top of the lineage tree in pseudotime, while those expressed in neurons such as *TP53I11* and *LMO3* are at the bottom, further suggesting a sequential production and high heterogeneity of subplate neurons in the GW10 cortex (Figure 3d,e). Moreover, SPN‐specific genes expressed in the RGCs, for instance *NR4A2* and *FBXW7* maintained stable expression levels, while those expressed in neurons such as *ST18* and *LMO3* showed increased expression levels along the developmental pseudotime (Figure S4f,g, Supporting Information). Early‐born neuron markers *TBR1* and *BCL11B* also displayed increased expression levels, and late‐born neuron markers *SATB2* and *CUX2* showed unchanged low expressions in GW10 cortices (Figure S4h,i, Supporting Information). These results further suggest that subplate neurons can be detected as early as GW10 in the human fetal cortex, and a specific RGC population can produce subplate neurons.^[^ ^25^ ^]^

Moreover, seven cell clusters including RGCs, IPCs, neurons, INs, and MG, with additions of oligodendrocyte precursor cells (OPCs) expressing *PDGFRA* and endothelial cells (ECs) expressing *IGFBP7* were annotated in the human fetal cortices at GW14 (Figure 3f,g; Figure S5a, Supporting Information). SPN‐specific genes such as *TLE4* and *FBXW7*, which were mainly detected in RGCs at GW10, were concentrated in the cluster of neurons at GW14, suggesting a directional differentiation of SPNs (Figure S5b, Supporting Information). A sequential differentiation trajectory, beginning at the RGCs and IPCs to neurons, which is similar to that in GW10 cortices, also was observed in the GW14 cortex (Figure 3h; Figure S5c, Supporting Information). A significant decrease of the percentage of RGCs from 64% to 16%, an expansion of IPCs from 5% to 10% and a dramatic production of neurons from 31% to 74% were detected when using Harmony to integrate datasets from the motor, somatosensory and V1 regions in GW14 cortices (Figure S5d, Supporting Information). These results suggest an increase of proportions of the IPC population and neurons in the human fetal cortex at GW14, compared to those at GW10.

Data integration and developmental trajectory analyses of the neuron clusters further showed three distinct subclusters with high expression of *EPHB6* ^+^/*SORBS2* ^+^, *SATB2* ^+^/*LMO4*,^+^ and *TBR1* ^+^/*VSNL* ^+^ (Figure 3i,j; Table S13, Supporting Information). Using the trajectory in pseudotime in the somatosensory region as a representative, we found that the *EPHB6* ^+^/*SORBS2* ^+^ subcluster reside at the root of the lineage tree, and is mixed with expression of IPC markers such as *PPP1R17* and *SSTR2*, they were thus named as newborn neurons (NBNs) in this study (Figure 3k; Figure S5e, Supporting Information). NBNs were split into two lineages that were both *SATB2*/*LMO4* positive. One population of *SATB2* ^+^/*LMO4* ^+^ cells (Lineage 2) further gave rise to *TBR1* ^+^/*VSNL* ^+^ cells with expression of SPN‐specific genes for instance *NR4A2* and *KCTD12* (Figure 3g,k). We thus named *SATB2* ^+^/*LMO4* ^+^ cells as migrating neurons (MGNs), and *TBR1* ^+^/*VSNL* ^+^ cells as subplate neurons (Figure 3j,k). Moreover, *TBR1*, *TLE4*, and *FBXW7* displayed an increased expression in the Lineage‐2, and *SATB2* showed an increase in the Lineage‐1, suggesting features of subplate neurons and late‐born neurons in the Lineage‐2 and Lineage‐1, respectively (Figure 3k,l). In addition, similar developmental trajectories in pseudotime of three subclusters were detected in the motor and V1 cortical regions using Monocle 2, indicating a common developmental theme in different regions in the human fetal cortex at GW14 (Figure S5f–i, Supporting Information).

In summary, our results of single‐cell RNA‐seq analyses suggest that some RGCs at GW10 and a lineage of migrating neurons at GW14 can produce subplate neurons in human fetal cortices.

### 2.4 Lineage Differentiation of Subplate Neurons

**Figure 4:**
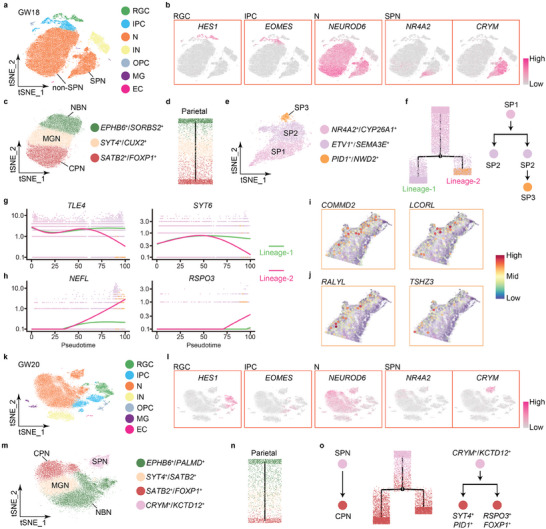
Lineage differentiation of subplate neurons in the human fetal cortices. a) Cell clusters of the human fetal cortex at GW18 based on single‐cell RNA‐seq datasets after Harmony integration depicted by t‐SNE. Each dot represents one cell. b) Known genes of RGCs, IPCs, neurons (N) and SPNs in the GW18 cortex visualized using t‐SNE. c) Cell subclusters of non‐SPN neurons of single‐cell RNA‐seq datasets of the GW18 cortex visualized by t‐SNE, with three subclusters such as newborn neurons (NBNs), migrating neurons (MGNs) and cortical plate neurons (CPNs). d) A lineage tree of NBNs, MGNs, and CPNs of single‐cell RNA‐seq datasets in the parietal region of the GW18 cortex. e) Three subclusters of subplate neurons including SP1, SP2, and SP3 in the GW18 cortex visualized by t‐SNE. f) A lineage tree of subplate neuron subtypes from the SP1 to SP3. g,h) Changes in expression levels of genes highly expressed in the Lineage‐1 (g) and Lineage‐2 (h) in the GW18 cortex. i,j) Spatial spots of expression patterns of representative new SPN genes such as *COMMD2* and *LCORL* annotated in the SP1 lineage and *RALYL* and *TSHZ3* in the SP2 in the cortical plate in the GW12 human fetal cortex. k) Cell clusters of the human fetal cortex at GW20 based on single‐cell RNA‐seq datasets. l) Known genes of RGCs, IPCs, neurons (N) and SPNs in the GW20 cortex visualized using t‐SNE. m) Cell subclusters of neurons of single‐cell RNA‐seq datasets of the GW20 cortex visualized by t‐SNE. n) A lineage tree of NBNs, MGNs and CPNs in the parietal region of the GW20 cortex. o) A lineage tree of subplate neurons to cortical plate neurons based on single‐cell RNA‐seq datasets of the GW20 cortex.

To track neuronal differentiation in the human fetal cortices at later stages, we analyzed single‐cell profiles of several regions in the cortex at GW18, and annotated 7 cell clusters including RGCs, IPCs, neurons, INs, OPCs, MG, and ECs (**Figure** **4**a,b; Figure S6a, Supporting Information). Among the neuron cluster, a clear subplate neuron subcluster and non‐SPN subcluster were observed, suggesting a unique trajectory separation of SPNs in the GW18 cortex (Figure 4a,b). Neurons in the non‐SPN subcluster were further divided into three subclusters: *EPHB6* ^+^/*SORBS2* ^+^, *SYT4* ^+^/*CUX2* ^+^, and *SATB2* ^+^/*FOXP1* ^+^ (Figure 4c; Table S14, Supporting Information). Focusing on the parietal region, the *EPHB6* ^+^/*SORBS2* ^+^ subcluster (NBNs) also was detected at the top of the lineage tree, the *SYT4* ^+^/*CUX2* ^+^ subcluster (MGNs) at the middle, and the *SATB2* ^+^/*FOXP1* ^+^ subcluster was at the bottom of the lineage by expressing *NEFL*, *FOXP1* and *RSPO3*, thus named as cortical plate neurons (CPNs) in the GW18 cortex (Figures 3, 4; Figure S6b,c, Supporting Information). The trajectories, which were detected in other cortical regions such as PFC, motor, somatosensory, and V1, were consistent with those in the parietal region, suggesting a common track of neuronal differentiation in the human fetal cortices at GW18 (Figure S6d,e, Supporting Information).

Interestingly, subplate neurons in the GW18 cortex also consisted of three subtypes, *NR4A2* ^+^/*CYP26A1* ^+^ (SP1), *ETV1* ^+^/*SEMA3E* ^+^ (SP2) and *PID1* ^+^/*NWD2* ^+^ (SP3), suggesting a heterogeneity of SPNs (Figure 4e; Table S15, Supporting Information). The SP1 neurons were separated into two subtypes in the developmental pseudotime, with the SP2 neurons in the Lineage‐1, and a small amount of SP2 and a large population of SP3 neurons in the Lineage‐2 constructed using Monocle 2 (Figure 4e,f). We compared expression levels of various marker genes in each lineage, and found that *TLE4* and *SYT6* are stably expressed in the Lineage‐1, while cortical plate neuron markers such as *NEFL* and *RSPO3* show increased expression in the Lineage‐2, suggesting that the SP3 subplate neurons display similarity to the CPNs (Figure 4g,h). Moreover, we incorporated in situ hybridization images of these genes for SPN subtypes based on Allen Brain Atlas, and observed their expression patterns in the cortical wall of brains at GW17‐18 (Figure S7, Supporting Information). These results suggest that while subplate neurons expand their own trajectory, they might also give rise to cortical plate neurons in the human fetal cortices at GW18.^[^ ^45^ ^]^

Moreover, we detected some new markers for subplate neurons by comparing genes annotated in the SPN subtypes such as SP1 and SP2 with reported SPN genes (Table S15, Supporting Information). To examine whether these genes might be expressed in human fetal cortices even at earlier stages, we selected representative genes *COMMD2* and *LCORL* annotated in the SP1 lineage and *RALYL* and *TSHZ3* in the SP2, and looked into their spatial spots in the S‐1 section collected from the GW12 cortex. These four new SPN markers were highly expressed in the cortical plate in the GW12 human fetal cortex (Figure 4i,j). In addition, we expanded our analyses and detected additional new SPN markers such as *GPR22*, *VSNL1*, and *CAMKV* with different expression levels in the cortical plate in the S‐1 and S‐2 sections in human fetal cortices at GW12 and GW13 (Figure S8, Supporting Information).

To follow further differentiation trajectories of SPNs, we annotated single‐cell RNA‐seq datasets in the GW20 cortex, detected similar cell clusters to those in GW18 cortices such as RGCs, IPCs and neurons, and neuronal subtypes for instance NBNs (*EPHB6* ^+^/*PALMD* ^+^), MGNs (*SYT4* ^+^/*SATB2* ^+^), CPNs (*SATB2* ^+^/*FOXP1* ^+^), and SPNs (*CRYM* ^+^/*KCTD12* ^+^), and observed a continuous trajectory from NBNs to MGNs and CPNs (Figure 4k–n; Figure S9a, Supporting Information). We extracted all genes expressed in the SPNs and CPNs to construct a lineage tree at GW20. We found that the *CRYM* ^+^/*KCTD12* ^+^ SPNs are split into two CPN lineages, with one expression of *SYT4* and *PID1* and one of *RSPO3* and *FOXP1*, which further suggest that some cortical plate neurons are differentiated from subplate neurons in the human fetal cortices at GW20 (Figure 4o).

Furthermore, we examined changes in gene expression levels in the subplate neuron population, and detected a significant decrease of expression of some SPN‐specific genes such as *NR4A2*, *ST18*, *FBXW7*, and *CRYM*, and an increase of IPC marker *SSTR2* and MGN marker *SYT4*, which was also annotated in the SPN subcluster, along the pseudotime in GW20 cortices (Figure S9b,c, Supporting Information). Meanwhile, the expression level of the early‐born neuron marker *TBR1* was decreased and that of the late‐born neuron marker for example *POU3F2* was increased (Figure S9d, Supporting Information). Moreover, *SSTR2* and *SYT4* showed continuous expressions in the *CRYM*‐positive SPN populations in the human fetal cortices from GW20 to GW25 (Figure S9e–g, Supporting Information). These results suggest that subplate neurons undergo a transition from possessing features of early‐born neurons to late‐born neurons from GW20 to GW25. In addition, percentages of SPNs in captured cells by single‐cell RNA‐seq declined gradually from 17.6% to 1.7% in the human fetal cortices from GW18 to GW25, further suggesting an active transition and a transient lineage of subplate neurons at fetal stages (Figure S9h, Supporting Information).

In summary, our single‐cell RNA‐seq results indicate a molecular heterogeneity of subplate neurons, and highlight a transition of SPNs from features of early‐born neurons to late‐born neurons and cortical plate neurons.

### 2.5 Maintaining Subplate Neurons by Human‐Specific Genes

**Figure 5:**
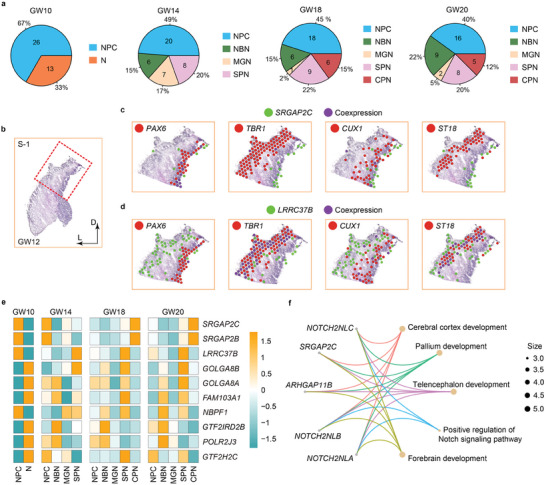
Maintaining subplate neurons by human‐specific genes. a) Numbers and percentages of human‐specific genes expressed in various cell types in human fetal cortices from GW10 to GW20, including neural progenitor cells (NPCs), neurons (N), newborn neurons (NBNs), migrating neurons (MGNs), subplate neurons (SPNs) and cortical plate neurons (CPNs). b‐d) The spatial spots of co‐expression of human‐specific genes *SRGAP2C* (c) and *LRRC37B* (d) with the NPC marker *PAX6*, early‐born neuron marker *TBR1*, late‐born neuron marker *CUX1*, and SPN‐specific gene *ST18* in the S‐1 section of the human fetal GW12 cortex. e) Heatmaps of expression levels of 10 representative human‐specific genes in various cell types in human fetal cortices from GW10 to GW20. f) Gene Ontology (GO) analysis of 68 human‐specific genes, adjusted *P* < 0.05.

Because of the critical role of subplate in the human brain, we examined whether genes evolutionarily known to be human‐specific might contribute to subplate neuron development.^[^ ^46^, ^47^ ^]^ We searched 68 reported human‐specific genes, compared their expressions in single‐cell RNA‐seq datasets, and detected 39, 41, 40, and 40 human‐specific genes expressed in the human fetal cortices at GW10, GW14, GW18, and GW20, respectively, suggesting that not all 68 human‐specific genes are continuously expressed in fetal cortices (Tables S16–S20, Supporting Information).^[^ ^48^, ^49^, ^50^, ^51^ ^]^ We calculated proportions of human‐specific genes expressed in various cells and found that 67% of them (26 genes) are highly expressed in neural progenitor cells (NPCs, including RGCs and IPCs) and 33% of them (13 genes) are expressed in neurons in the GW10 cortex (**Figure** **5**a). The proportions of human‐specific genes expressed in NPCs gradually decreased to 40% in the GW20 cortex, while those expressed in distinct neurons increased (Figure 5a). Interestingly, the proportions (20%) of human‐specific genes expressed in subplate neurons in the human fetal cortices remained stable from GW14 to GW20, in particular, *GOLGA8A*, *GOLGA8B*, *LINC00957*, *LRRC37B*, *RASA4B*, and *PTPN20* were detected in SPNs in cortices at least at two developmental stages (Figure 5a; Tables S18–S20, Supporting Information). These results suggest that human‐specific genes might play a role in maintaining the population of subplate neurons in human fetal cortices.

To visualize expression patterns of human‐specific genes in the cortex, we selected *SRGAP2C* and *LRRC37B*, and looked into their spatial expression spots at early stages based on spatial transcriptomics (Figure 5b–d). We co‐localized both genes with spatial spots of the NPC marker *PAX6*, early‐born neuron marker *TBR1*, late‐born neuron marker *CUX1* and SPN‐specific gene *ST18* in the S‐1 section of the GW12 cortex (Figure 5b–d). *SRGAP2C* was largely co‐expressed in the *PAX6* ^+^ progenitor cells, with scattered expression in *CUX1* ^+^ cells, suggesting its expression in NPCs (Figure 5b,c). *LRRC37B* was mainly co‐expressed with *TBR1* and *ST18*, indicating its expression in subplate neurons (Figure 5b,d). These spatial‐transcriptomic results demonstrate that human‐specific genes are expressed in distinct cell types in the human fetal cortices.

Furthermore, we compared expression levels of human‐specific genes expressed in human fetal cortices from GW10 to GW20, extracted 10 representative genes, and analyzed their expressions in various cell types (Figure 5e). We found that they display distinct and dynamic expression patterns, for instance, *SRGAP2B* and *SPGAP2C* are highly expressed in NPCs in GW10 and GW14 cortices, and then in differentiated CPNs in GW18 and GW20 cortices (Figure 5e). Genes such as *POLR2J3* and *GTF2IRD2B* displayed stable expression levels in NBNs at all stages, suggesting their role in maintaining newborn neuron populations. Moreover, to explore functions of human‐specific genes, we performed Gene Ontology (GO) analysis of all 68 human‐specific genes and found that *NOTCH2NLA*, *NOTCH2NLB*, *NOTCH2NLC*, *ARHGAP11B* and *SRGAP2C* are hub genes involved in multiple processes, such as pallium development and telencephalon development (Figure 5f).

In summary, our single‐cell RNA‐seq and spatial transcriptomics results indicate that human‐specific genes maintain the subplate neuron lineage, and are likely driving forces for neuronal differentiation in the process of human fetal brain development.

### 2.6 Cortical Regional Enrichment of ECM Genes in Subplate Neurons

**Figure 6:**
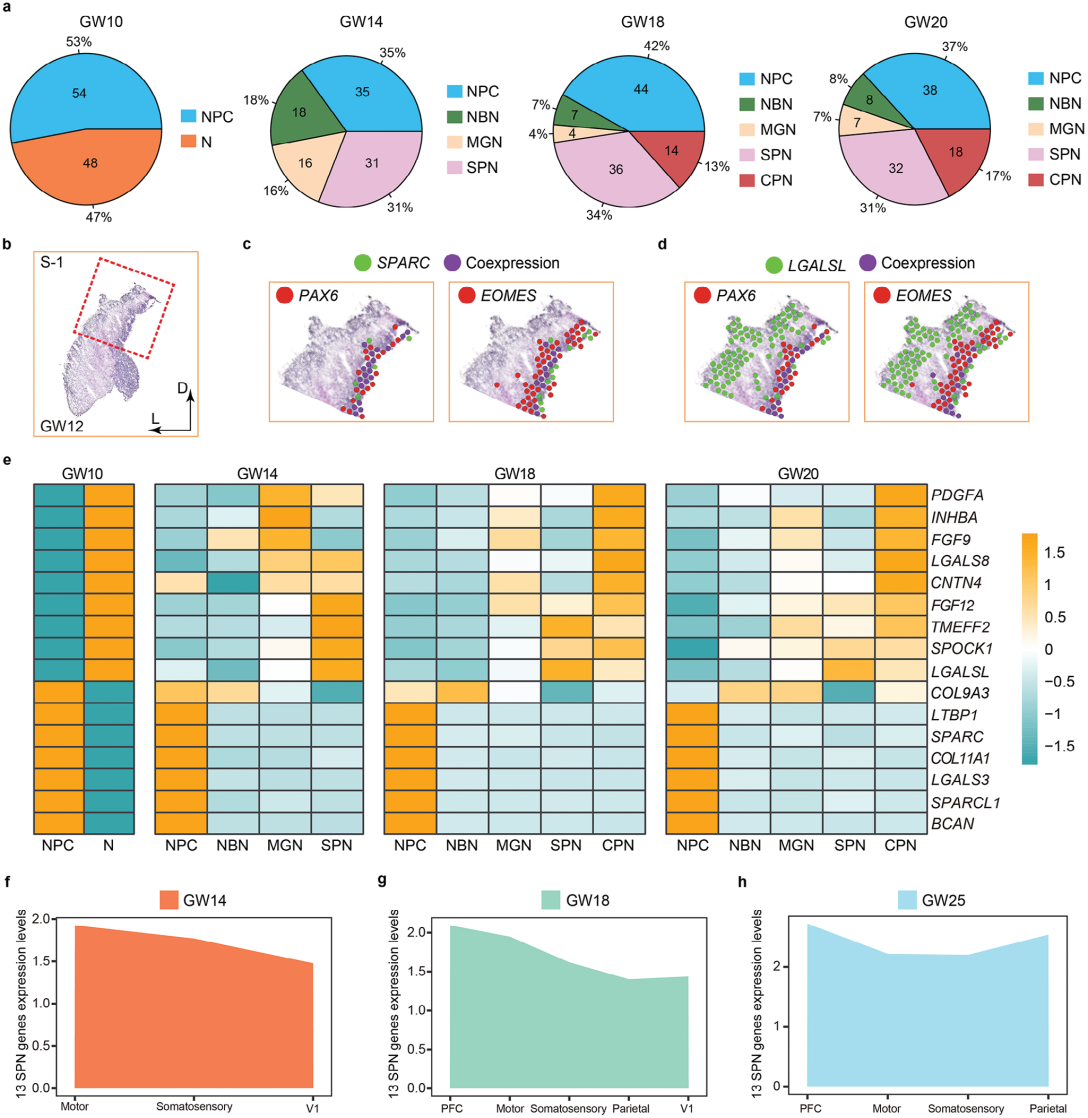
Cortical regional enrichment of extracellular matrices (ECMs) genes in subplate neurons. a) Numbers and percentages of ECM genes expressed in various cell types in human fetal cortices from GW10 to GW20. b‐d) The spatial spots of co‐expression of the ECM genes *SPARC* (c) and *LGALSL* (d) with the RGC marker *PAX6* and IPC marker *EOMES* in the S‐1 section of the human fetal GW12 cortex. e) Heatmaps of expression levels of 16 representative ECM genes in various cell types in human fetal cortices from GW10 to GW20. f‐h) Regional area plots of the sum of expression levels of 13 ECM genes highly expressed in subplate neurons in each cortical regions in the GW14, GW18, and GW25 human fetal cortices.

Previous studies have shown that accelerated neurogenesis including neural progenitor cells and neurons, in particular subplate neurons, contributes to cortical folding.^[^ ^35^, ^52^ ^]^ Because the subplate is abundant with extracellular matrices (ECMs) and components of ECM appear to have a significant impact on cortical convolution,^[^ ^32^, ^33^, ^53^, ^54^, ^55^, ^56^ ^]^ we analyzed single‐cell RNA‐seq datasets, and captured 103 ECM genes expressed in NPCs and neurons in the GW20 cortex, based on 108 reported ECM genes (Table S21, Supporting Information).^[^ ^36^ ^]^ In particular, 102, 100, 105, and 103 ECM genes were detected in the human fetal cortices at GW10, GW14, GW18, and GW20, respectively (Tables S22–S25, Supporting Information). We compared 108 reported ECM genes, among which 44 genes were highly expressed in the human cortex, 58 genes were highly expressed in the rodent cortex, and 6 genes were expressed in both humans and rodents, suggesting that some ECM genes might be human‐specific (Table S26, Supporting Information). We calculated proportions of ECM genes expressed in various cell types in cortices from GW10 to GW20 (**Figure** **6**a). Large proportions of ECM genes were highly expressed in cortical NPCs at all stages. Noticeably, the proportions of ECM genes detected in subplate neurons maintained stable growth (31% to 36%) in human fetal cortices from GW14 to GW20 (Figure 6a). These results suggest an important role of ECM genes in development of neural progenitors and subplate neurons.

We selected 4 ECM genes *SPARC*, *COL11A1*, *LGALSL*, and *SPOCK1*, and examined their expression patterns in the GW12 cortex based on spatial transcriptomic datasets (Figure 6b; Figure S10a,b, Supporting Information). We co‐localized them with expression spots of the NPC markers *PAX6* and *EOMES*. We found that *SPARC* and *COL11A1* are highly co‐expressed with *PAX6* and *EOMES*, while *LGALSL* and *SPOCK1* are mostly expressed in subplate, supporting their cell type specific spatial distribution in the human fetal cortex (Figure 6c,d; Figure S10a,b, Supporting Information).

Moreover, we identified differentially expressed ECM genes in NPCs and neurons, and detected 6 ECM genes, for instance *BCAN*, *SPARCL1*, and *LTBP1* with high expression levels in NPCs, and 10 ECM genes such as *FGF12*, *TMEFF2*, and *PDGFA* in neurons in the human fetal cortex at GW20 (Tables S27 and S28, Supporting Information). These 6 ECM genes expressed in NPCs displayed high expression levels in the human fetal cortices at all stages, suggesting their roles in maintaining the neural progenitor pool (Figure 6e; Table S29, Supporting Information). The other 10 ECM genes expressed in neurons displayed dynamic levels in distinct neuronal subtypes during differentiation (Figure 6e; Table S29, Supporting Information). We applied GO analyses to analyze the biological processes (BP), cellular components (CC), and molecular functions (MF) of these 16 representative ECM genes, and found that they are mainly involved in activin receptor signaling pathway, extracellular matrix binding, growth factor activity and receptor ligand activity, suggesting the role of these ECM genes in regulating cell proliferation and differentiation (Figure S10c, Supporting Information).

In addition, to explore the active regions of subplate expansion and folding at different stages, we identified 13 ECM genes with continuously high expression in SPNs from GW14 to GW20, such as *SCUBE3*, *BMPER*, and *LGALSL*, and calculated their expression levels in SPNs in each cortical region from GW14 to GW25 (Tables S23–S25 and Tables S30–S34, Supporting Information). We plotted regional area maps for the sum of expression levels of these 13 ECM genes expressed in SPNs in each region, and detected the highest level in the prefrontal and motor cortical regions, suggesting that enrichment of ECM genes in subplate neurons is more prominent in the frontal lobe than other regions in the human fetal cortices from GW14 to GW25 (Figure 6f–h). Similarly, we identified 21 ECM genes that maintained high expression in NPCs from GW14 to GW20 such as *BCAN*, *HAPLN1* and *LUM* (Tables S23–S25, Supporting Information). We found that the sums of expression levels of these 21 ECM genes are higher in the somatosensory cortical region at GW14, in the V1 region at GW18, and in the motor region at GW25 than other cortical regions (Figure S10d–f and Tables S35–S40, Supporting Information). In addition, we searched further current reports of ECM gene expression in the developing cortices, and did not detect ECM genes that showed cortical areal specific expression patterns in general.^[^ ^32^, ^33^, ^53^, ^54^, ^55^, ^56^ ^]^

In summary, our single‐cell RNA‐seq and spatial transcriptomics results indicate an important role of ECM genes in maintaining cortical subplate neurons. These results further show that enrichments of ECM genes in neural progenitors and subplate neurons are different in distinct cortical regions, suggesting existing of regional specific expansion of NPC and SPN populations in the human fetal cortices.

## 3 Discussion

Subplate neurons are one of the earliest born neurons in the human fetal cortex.^[^ ^11^, ^12^, ^24^ ^]^ They are involved in establishing topographic cortical connections through receiving corticofugal and thalamocortical axonal inputs during early development.^[^ ^11^, ^27^ ^]^ Due to the fast size‐expansion and limited tissue availability of the fetal human brains, molecular characteristics and developmental processes of subplate neurons remain unclear. In this study, we have revealed molecular features of the origin and lineages of subplate neurons in human fetal cortices from GW10 to GW25 by employing spatial transcriptomic technology and single‐cell RNA sequencing. Our study has generated molecular landscapes of subplate neurons, which should serve a better understanding of fundamental features of human fetal brain development.

In the process of human cortical neurogenesis, expansion of neural progenitors and generation and migration of differentiated neurons assemble a convoluted multiple layered human brain structure.^[^ ^2^, ^57^ ^]^ In this study, by applying spatial transcriptomic technology, we are able to visualize spatial and lineage distributions of various markers for RGCs in the VZ, IPCs in the SVZ, and neurons in the IZ and the CP in the human fetal cortical wall, by expressing *PAX6*, *EOMES*, and *NEUROD2*, respectively. Moreover, our results have revealed precise positional information of specific markers for subplate neurons such as *NR4A2*, *ST18*, and *ZFPM2* in the cortex. In addition, we also have detected a population of microglial cells positioned at the pallial/sub‐pallial boundary in the human cortex at GW12. Whether they function in early axonal guidance, synaptogenesis or neuronal cell death in the human fetal brain requires further research in future works. In summary, our findings highlight a significant implication of spatial transcriptomics in uncovering human brain temporal and spatial complicity in fetal stages.

Subplate neurons are among the earliest born cortical neurons.^[^ ^11^, ^12^, ^24^ ^]^ SPNs have been detected in the human fetal cortices from GW8 to GW42 or from GW17 to GW37, depending on the methods of examinations and human brain tissue availability.^[^ ^4^, ^11^, ^15^, ^16^, ^17^ ^]^ The earliest fetal stage that we analyzed in this study is GW10. Although we didn't annotate a unique cell cluster of SPNs in the GW10 cortex based on single‐cell RNA‐seq datasets, we observed expression of some markers for SPNs such as *NR4A2* and *CYP26A1* in the RGC cluster, and SPN markers such as *ST18* and *SLC12A5* in the neuron cluster. These findings indicate that the emerging time of human SPNs can be at least as early as GW10, and some RGCs can generate SPNs. Because the human cortex is at active stages of transitioning from the presubplate to the subplate up to GW15, our results are consistent with previous findings, in which cells expressing subplate markers are positioned near or within the CP at stages of GW12‐15.^[^ ^18^, ^20^, ^25^, ^44^ ^]^

In this study, we have tracked molecular trajectories of SPNs in the human fetal cortices by focusing on revealing their developing processes from GW14 to GW20. We have found that markers for SPNs are exclusively detected in cell clusters of neurons, no longer in those of neural progenitors, suggesting a differentiation time point of SPNs at GW14 in human fetal cortices. Moreover, according to our developmental trajectory study, SPNs are derived from subclusters of newborn neurons (NBNs) and subsequently migrating neurons (MGNs), indicating a sequential generation and differentiation of SPNs. Noticeably, we have compared gene expression patterns of SPNs with those of early‐born neurons such as *TBR1* and *BCL11B*, and have found a significant co‐expression of genes in both neuronal populations, suggesting that subplate neurons possess the molecular characteristics of early‐born neurons in the cortices from GW12 to GW14.

Moreover, we have detected a molecular diversity of subplate neurons by expression of distinct combinations of unique genes in the GW18 cortex. Our study of single‐cell RNA‐seq further supports the molecular heterogeneity of SPNs, which have been proposed in previous studies.^[^ ^21^, ^22^, ^23^, ^24^, ^25^ ^]^ We also have noticed a molecular transition of subplate neurons from features of early‐born neurons to late‐born neurons, by increased co‐expression of late‐born neuron markers such as *POU3F2* in the SPN population in the GW18 cortex. A subtype of subplate neurons (SP1) was grouped into a subcluster containing proliferative cells, likely neural progenitors, which produce more mature SPNs at GW18 (Figure 4). These SPNs (SP1) also can give rise to differentiated cortical plate neurons, suggesting that SPNs are an important source of cortical neurons during neurogenesis.^[^ ^21^, ^22^, ^25^ ^]^

In addition, because the subplate is a transient layer in the developing cortex, one expects to observe a significant increase as well as decrease of proportions of SPNs over time. Indeed, we have found a continuously decreased population of SPNs in the human fetal cortices from GW18 to GW25, which is within the period of SPN development from GW17 to GW37 observed by others.^[^ ^11^, ^15^, ^16^, ^17^ ^]^ Differentiation of SPNs to other cortical neurons contributes to the transient changes of the SPN population.^[^ ^18^, ^20^, ^25^, ^44^ ^]^

Moreover, we have detected cohesive patterns of molecular trajectories of the SPN lineage and population changes of SPNs in various cortical regions from GW18 to GW20, for instance in the somatosensory region, and in the motor and V1 regions. These results indicate a common developmental theme of SPN development in the human fetal cortex. The precise generation and trajectory formation of SPNs, shown in this study, further support the instructive role of SPNs in thalamocortical and intracortical connectivity at critical stages of human fetal brain development.^[^ ^19^, ^27^, ^30^, ^31^ ^]^ Future works may investigate what might trigger these molecular programs and cascades to make the subplate a transient structure in the cortex.

Previous studies have shown that even though the subplate is an evolutionally conserved structure, it still expresses a unique set of genes in the human fetal cortices.^[^ ^22^, ^58^, ^59^ ^]^ We have thus examined expression patterns of human‐specific genes in the SPN population.^[^ ^48^, ^49^, ^50^, ^51^ ^]^ We have detected a stable proportion of SPNs with high expression levels of human‐specific genes from GW14 to GW20. These results indicate that human‐specific genes, as a driving force of evolution of human cortical development and expansion, likely play a role in maintaining the SPN population, which further hints that they might contribute to the unique feature of SPNs in the human fetal brain compared to other species.

Furthermore, because the subplate is abundant with extracellular matrices (ECMs), we examined expression profiles of ECM genes in the human fetal SPNs in this study.^[^ ^32^, ^33^ ^]^ Similar to the human‐specific genes, ECM genes also were expressed in SPNs with stable proportions in the human fetal cortices from GW14 to GW20, suggesting their functions in maintaining SPNs. Interestingly, we have detected enriched ECM genes in SPNs positioned in the frontal lobe in the human fetal cortices. Enriched and regional expression of ECM genes appear to be cell type specific, for instance in subplate neurons observed in this study, since we have not detected ECM genes that show cortical areal specific expression patterns in general based on current published reports.^[^ ^32^, ^33^, ^53^, ^54^, ^55^, ^56^ ^]^ Moreover, we have detected 44 ECM genes that are highly expressed in the human cortex, suggesting that some ECM genes might be human‐specific. Taking into consideration the potential roles of ECM genes in cortical folding, and SPN contribution to the convolution of human fetal cortices,^[^ ^34^, ^35^, ^36^ ^]^ the enrichment of ECM genes in the SPN population in the frontal lobe might be associated with brain structural formation. Whether these ECM genes may preferentially affect SPN expansion and how this regional effect might contribute to cortical folding are intriguing directions for future studies.

In summary, our work has revealed the molecular characteristics and origin of human fetal subplate neurons, and defined their different subclusters and developmental trajectories. Our study should have generated a rich source to further uncover the enigma of the subplate development in the human fetal brain.

## 4 Experimental Section

### Ethical Statement

This study was approved by the Ethical Committee of the Quanzhou First Hospital (20162016). Human fetal brain tissue samples were collected from elective pregnancy termination specimens at the Quanzhou First Hospital, Fujian Province, in China. Discarded human tissue samples were examined only from patients who had given informed consent with no compensation. All the protocols complied with the “Interim Measures for the Administration of Human Genetic Resources”.

### Frozen Embedded Tissue Samples for Spatial Transcriptomics

Discarded human fetal tissue samples were collected at GW12 and GW13, one brain at each stage. Staging of fetuses was followed as described by Hern.^[^ ^60^ ^]^

The fresh tissue surfaces were quickly rinsed with a pre‐cooled solution of 1× PBS (RNase free) or normal saline to remove residual blood, and sterile gauze was used to blot the surface fluid. The cerebral cortex was selected from each tissue sample at different stages, and was cut into small pieces (6.5 mm^3^) suitable for subsequent experiments. Small pieces of the cortex from each tissue sample were snap‐frozen in isopentane pre‐chilled with liquid nitrogen and optimum cutting temperature (OCT) compound (SAKURA, Cat#: 4583), and stored at −80 °C until use.

The tissue quality inspection of the cortex was carried out by cutting 10 sections of each embedded tissue (the thickness of the tissue section was 10 µm) for RNA quality inspection, and RIN ≥ 7.0 was required. Only the qualified cortical tissues were used to perform tissue permeability and gene expression experiments.

### Spatial Transcriptomics Slides Preparation

Slides for spatial transcriptomics were printed with four identical 6.5 × 6.5 mm capture areas, each with 5000 spots containing barcoded primers (10x Genomics). The primers were attached to the slide by the 5′ end and contained a cleavage site, a T7 promoter region, a partial read1 Illumina handle, a spot‐unique spatial barcode, a unique molecular identifier (UMI), and a poly(dT)VN. The spots had a diameter of 55 µm and were arranged in a centered and regular hexagonal grid so that each spot had six surrounding spots with a center‐to‐center distance of 110 µm. Each frozen tissue sample was cut in a pre‐cooled cryostat at 10 µm thickness and systematically placed on chilled Visium Tissue Optimization Slides (3000394,10x Genomics) and Visium Spatial Gene Expression Slides (2000233, 10x Genomics), and stored at −80 °C until use.

### Further optimization of tissue samples for spatial transcriptomics

The spatial transcriptomics (ST) protocol was optimized for tissue according to recommendations.^[^ ^61^ ^]^ In short, changes were made in the staining procedure by excluding isopropanol, decreasing the incubation time of hematoxylin and bluing buffer, as well as increasing eosin concentration. Moreover, the previously described one‐step protocol for tissue removal was altered by using a higher proteinase K:PKD buffer ratio, and the optimal incubation time for permeabilization was established based on the fluorescence intensity generated by different permeabilization times. Once optimal conditions had been established, three cryosections per cortical tissue sample were cut at 10 mm thickness onto spatial slides and processed immediately.

### Fixation, Staining, and Imaging

Sectioned slides were incubated at 37 °C for 1 min, fixed in 3.7%–3.8% formaldehyde (Sigma‐Aldrich) in PBS (Medicago) for 30 min, and then washed in 1× PBS (Medicago).

For staining, sections were incubated in Mayer's hematoxylin (Dako, Agilent, Santa Clara, CA) for 4 min, bluing buffer (Dako) for 30 s, and Eosin (Sigma‐Aldrich) diluted 1:5 in Tris‐base (0.45 M Tris, 0.5 M acetic acid, pH 6.0) for 30 s. The slides were washed in RNase and DNase free water after each of the staining steps.

After air‐drying, the slides were mounted with 85% glycerol (Merck Millipore, Burlington, MA) and coverslips (Menzel‐Glaser). Bright‐field (BF) images were taken at 20× magnification using Metafer slide scanning platform (MetaSystems). Raw images were stitched with VSlide software (MetaSystems). The coverslip and glycerol were removed after imaging by immersing slides in RNase and DNase free water.

The slides were inserted into slide cassettes to separate the tissue sections into individual reaction chambers (hereinafter wells). For pre‐permeabilization, sections were incubated at 37 °C for 20 min with 0.5 U mL^−1^ collagenase (ThermoFisher) and 0.2 mg mL^−1^ BSA (NEB, Ipswich, MA) in HBSS buffer (ThermoFisher). Wells were washed with 0.1× SSC (Sigma‐Aldrich), after which permeabilization was conducted at 37 °C for 7 min in 0.1% pepsin (Sigma‐Aldrich) dissolved in 0.1 M HCl (Sigma‐Aldrich). After incubation, the pepsin solution was removed, and wells were washed with 0.1× SSC.

### Reverse Transcription, Spatial Library Preparation, and Sequencing

Reverse transcription (RT) was conducted as previously described.^[^ ^61^ ^]^ After RT, wells were washed with 0.1× SSC and incubated at 56 °C with interval shaking for 1.5 h with a tissue removal mix of Proteinase K (QIAGEN) and PKD buffer (QIAGEN, pH 7.5) at a 1:1 ratio. The spatially barcoded cDNA was enzymatically released as previously described.^[^ ^61^ ^]^ Supernatants containing released cDNA were collected and transferred to 96‐well plates for RT library preparation with an automated MBS 8000 system.^[^ ^62^ ^]^ In short, second‐strand cDNA synthesis was followed by in vitro transcription, adaptor ligation, and a second RT. Sequencing handles and indexes were added in an indexing PCR and the finished libraries were purified and quantified as previously described.^[^ ^63^ ^]^ Sequencing was performed on the Illumina NovaSeq 6000 with a sequencing depth of at least 50 000 reads per spots and 150 bp (PE150) paired‐end reads (performed by Biomarker Technologies Corporation, Beijing, China).

### Spot Visualization and Image Alignment

The spot staining and imaging procedure were described previously.^[^ ^61^ ^]^ In short, primer spots were stained by hybridization of fluorescently labeled probes and imaged on the Metafer slide scanning platform. The resulting spot image was loaded into the web‐based ST spot detector tool along with the previously obtained BF tissue image of the same area.^[^ ^64^ ^]^ The two images were aligned and the built‐in tissue recognition tool was used to extract spots covered by tissue.

### Spatial Transcriptomics Data Upstream Analysis

Finished libraries were diluted to 4 nM and sequenced on the Illumina Nova 6000 using paired‐end sequencing. The upstream analysis was completed through Space Ranger (v2.0.0). The mapping was performed to the reference GRCh38_release95 human genome.

### Clustering, Annotation, and Gene Expression of Spatial Transcriptomic Data

The 1162 spatial points under the cortical tissue were all applied to the downstream analysis. There were some differences in sequencing depth between spatial points, so it was necessary to standardize the data. “SCTransform” function was used to normalize the data. 3000 high variance features were detected and stored the data in SCT analysis. Principal component analysis (PCA (“RunPCA” function, using variable genes from SCT analysis)) and graph‐based Louvain (“FindNeighbors” and “FindClusters”, data dimensionality reduction using PCA, the PCA number was 30, and the clustering resolution was 1.5) were used for dimensionality reduction and clustering of the data.

The differentially expressed genes (DEGs) were computed using the “FindAllMarkers” function (default parameters) and the genes with log_2_ fold change > 0.25 and adjusted *P* < 0.05 were selected as marker genes of cell clusters. Four major clusters were identified and annotated as RGCs, IPCs, neurons, and MG based on the marker genes of cell clusters and the spatial expression of feature genes of specific cell types (such as *PAX6* and *HES1* in RGCs). IPCs have 2 subclusters (IPC1 and IPC2) and neurons have 8 subclusters (N1‐N8). Visualization was performed using UMAP (“RunUMAP” function) to present data in 2D coordinates.

The spatial expression profiles of the target genes were constructed by the “SpatialFeaturePlot” function (alpha = c(0.1, 1)).

### Pearson Correlation Coefficient Between Different Cell Clusters

The “AverageExpression” function was used to calculate the expression levels of all genes in IPC1, N1 to N4 from GW12 (S‐1) and IPC2, N5 to N8 from GW13 (S‐2, S‐3, and S‐4), respectively. The significantly expressed genes (log_2_ fold change > 0.25, pct.1 > 0.6, pct.2 < 0.4, and adjusted *P* < 0.05, mitochondrial related genes and ribosome related genes were removed) of these 10 cell clusters were obtained by using the “FindAllMarkers” function (default parameters), and the expression levels of these significantly expressed genes were screened from the expression levels of all genes in each cluster.

The Pearson correlation coefficient matrix between the 10 cell clusters was calculated by the “rcorr” function based on the expression levels of the significantly expressed genes in each cell cluster. Cell clusters with strong correlation (correlation coefficient between cell clusters ≥ 0.7 and *P* < 0.05) were marked with asterisks (*).

### Expression Heatmaps of 8 SPN Genes in Intermediate Progenitor Cells and Neurons at GW12 and GW13

Among 124 reported SPN markers,^[^ ^22^ ^]^ 8 were highly expressed in S‐1 at GW12 (pct.1 > 0.6 and pct.2 < 0.4), and 19 were highly expressed in S‐2 to S‐4 sections at GW13 (Tables S6,S8, Supporting Information). Among these 19 SPN genes, 15 were significantly expressed in the N7 cluster and 4 were highly expressed in both N7 and N8 clusters (Table S8, Supporting Information). *NSF1*, *CHPT1*, *HS3ST4*, *FBXW7* were selected with higher expression levels in the S‐1 at GW12, and *ZFPM2*, *ST18*, *GPD1L*, *NLGN1*, which are highly expressed in both N7 and N8 clusters in S‐2 to S‐4 sections at GW13, and then constructed their expression heatmaps in intermediate progenitor cells and neurons from GW12 and GW13.

### Constructing Developmental Ridge Plots of Cells from Spatial Transcriptomics Data

Taking GW12 cell clusters as an example, the “FindAllMarkers” function was used to find significant DEGs (log_2_ fold change > 0.25, pct.1 > 0.6, pct.2 < 0.4, and adjusted *P* < 0.05) between cell clusters. After removing mitochondrial genes, ribosomal genes and repetitive DEGs, an ideal gene list was obtained. Using Monocle 2 to generate a data object for GW12 cell clusters, embedding the obtained gene list into it and using “DDRTree” for dimensionality reduction to obtain the pseudotime developmental trajectory of the cell clusters.^[^ ^65^ ^]^ “geom_density_ridges” function was used to reconstruct the trajectory to generate the ridge plot of cell development.

The construction of the developmental ridge plot of GW13 cell clusters was similar, with only slight differences in the conditions for identifying significant DEGs (log_2_ fold change > 0.25, pct.1 > 0.6, pct.2 < 0.3, and adjusted *P* < 0.05) in the cell clusters.

### 10x Genomics Single‐Cell RNA Sequencing Capture and Processing

Brain tissue samples were collected from GW10 to GW25 (GW10, GW14, GW18, GW20, GW22 and GW25).^[^ ^39^ ^]^ Staging of fetuses followed the procedure as described by Hern.^[^ ^60^ ^]^ Brain dissections were performed under a stereoscope with regards to major sulci to identify cortical regions. Tissue was incubated in 4 mL of papain/DNAse solution (Worthington) for 20 min at 37 °C, after which it was carefully triturated with a glass pipette, filtered through a 40‐µm cell strainer, and washed with HBSS. The GW22 and GW25 samples were additionally passed through an ovomucoid gradient (Worthington) in order to minimize myelin debris in the captures. The final single‐cell suspension was loaded onto a droplet‐based library prep platform Chromium (10x Genomics) according to the manufacturer's instructions. cDNA libraries were quantified using an Agilent 2100 Bioanalyzer and sequenced with an Illumina NovaSeq S4.

### Quality Control, Doublet Removal, and Cell Cycle Analysis in Single‐Cell RNA‐Seq Datasets

Publicly available datasets of various regions from the human fetal cortex were employed (https://data.nemoarchive.org/biccn/grant/u01_devhu/kriegstein/transcriptome/scell/10x_v2/human/processed/counts/).^[^ ^39^ ^]^ Cell count matrices in eight regions from GW10 to GW25 (GW10, GW14, GW18, GW20, GW22, and GW25) were collected for analysis. Quality control was performed to eliminate low‐quality cells. The gene number, total RNA number, mitochondrial gene percentage, ribosomal gene percentage, and red blood cell gene percentage of each cell in all regions at each stage were calculated. Cells with mitochondrial gene percentage higher than 15%, ribosomal gene percentage higher than 40%, and red blood cell gene percentage higher than 10% were excluded. Due to the differences in the number of genes and total RNA at each stage of cells, the screening of cells at each stage had different schemes.

At GW10, cells with gene numbers in the range of 200 to 6000 and total RNA numbers less than 6000 were retained. At GW14, cells with gene numbers in the range of 200 to 4000 and total RNA numbers less than 20 000 were retained. At GW18, cells with gene numbers in the range of 200 to 6000 and total RNA numbers less than 30 000 were retained. At GW20, cells with gene numbers in the range of 200 to 7500 and total RNA numbers less than 50 000 were retained. At GW22 and GW25, cells with gene numbers in the range of 200 to 6000 and total RNA numbers less than 20000 were retained. After cell screening, mitochondrial related genes, ribosomal related genes, red blood cell related genes (*HBA1*, *HBA2*, *HBB*, *HBD*, *HBE1*, *HBG1*, *HBG2*, *HBM*, *HBQ1*, and *HBZ*), and sex related genes (*DDX3Y*, *EIF2S3Y*, *UTY*, *KDM5D*, *XIST* and *TSIX*) were all removed for downstream analysis.

Doublets were abducted by Doublet Detection (https://github.com/JonathanShor/DoubletDetection) using default parameters.^[^ ^66^ ^]^ After quality control and removal of doublet cells, 241 979 high‐quality cells were selected from a total of 314 889 cells. There were 6643 cells remaining from the original 7194 cells at GW10, 37638 cells remaining from the original 43 555 cells at GW14, 55823 cells remaining from the original 62 768 cells at GW18, 35868 cells remaining from the original 41 113 cells at GW20, 68082 cells remaining from the original 117 482 cells at GW22 and 37 925 cells remaining from the original 42777 cells at GW25. To remove the effect of cell cycle on cell clustering and dimensionality reduction, Seurat packages, containing a cell‐cycle‐related gene set with 43 genes for the G1/S phase and 54 genes for the G2/M phase of the cell cycle, were applied.^[^ ^67^ ^]^ The G1/S and G2/M states of each cell were defined using the “CellCycleScoring” function, and the differences between the G2 M and S phase scores were regressed out using the “ScaleData” function.

### Clustering and Annotation

The downstream analysis was performed using data normalization (“NormalizeData” function, “LogNormalize” method and scaling factor 10000), data feature scaling (“ScaleData” function eliminated the impact of cell cycle on clustering), variable gene detection (“FindVariableFeatures” function, top 2000 genes with the highest standardized variance selected using the “vst” selection method), PCA (“RunPCA” function, from 2000 variable genes) and Louvain graph‐based clustering (“FindNeighbors” and “FindClusters”, data dimensionality reduction using PCA. The PCA number was 30, dimension of reduction was 30 and the clustering resolution was 0.6). The top 30 significant principal components (PCs) were selected to alleviate technical variation between different batches by using the R package Harmony,^[^ ^68^ ^]^ using the “FindAllMarkers” function (thresh.use = 0.25 and test.use = “bimod”) with the Seurat R package to compute DEGs (log_2_ fold change > 0.25 and adjusted *P* < 0.05) in cell clusters at each stage. By using known marker genes to assign the identity of the cell clusters and then merging cell clusters of the same type. Seven clusters were identified and annotated as RGCs, IPCs, neurons (Ns), INs, OPCs, MG, and ECs from single‐cell RNA‐seq datasets at GW10‐GW20. Visualization was performed using t‐SNE to present datasets in 2D coordinates, generated by the “RunTSNE” function, and t‐SNE plots were generated using the R package “ggplot2”.

Cell clusters of neurons at GW14 were extracted for further analysis. According to the DEGs of each cell type, cell clusters with similar gene expression in neurons at GW14 were merged. DEGs (log_2_ fold change > 0.25 and adjusted *P* < 0.05) were recalculated and it was found that neurons can be divided into three cell types: NBNs, MGNs, and SPNs.

The neuron cluster at GW18 was divided into two parts for analysis based on DEGs in cell clusters and the visualization of t‐SNE. According to the DEGs of each cell cluster at GW18, neurons in the non‐SPN subcluster were further divided into three subclusters NBNs, MGNs, and CPNs, and the DEGs (log_2_ fold change > 0.25 and adjusted *P* < 0.05) between them were recalculated using the “FindAllMarkers” function (thresh.use = 0.25 and test.use = “bimod”). SPNs were also divided into three subclusters SP1, SP2, and SP3. Since the genes expressed between these three subclusters were similar, the screening criteria of DEGs was improved (log_2_ fold change > 0.25, pct.1 > 0.25, pct.2 < 0.2, and adjusted *P* < 0.05) and selected the markers of SP1, SP2, and SP3 from the screened genes.

### Trajectory and lineage Analysis

Monocle 2 was used to construct various pseudotime developmental trajectories and lineage trees of cell clusters from GW10, GW14, GW18, and GW20, and the process was almost consistent at each stage.^[^ ^65^ ^]^ Cell clusters needed to construct cell trajectories or lineage trees were extracted and their DEGs (log_2_ fold change > 0.25 and adjusted *P* < 0.05) were calculated using the “FindAllMarker” function (thresh.use = 0.25 and test.use = “bimod”). Then, ideal gene lists were selected from the DEGs, embedded into the data objects generated by Monocle 2 and dimensionality was reduced using “DDRTree”. The developmental trajectories of the data objects were visualized using the “plot_cell_trajectory” function and the lineage trees of the data objects using the “plot_complex_cell_trajectory” function.

The selection criteria of the gene list used for the developmental trajectory or lineage tree of each data object were different. Screening the gene list from DEGs of RGCs, IPCs, and neurons from PFC, central, and occipital regions at GW10 was done according to pct.1 > 0.24, pct.2 < 0.6 and was excluded duplicate genes. It was consistent with the conditions for screening of gene list from the DEGs of RGCs, IPCs, and neurons expressing 14 SPN specific genes from PFC, central, and occipital regions at GW10. Pct.1 > 0.13, pct.2 < 0.86 were required to screen the gene list from DEGs of RGCs, IPCs, and neurons in the somatosensory region at GW14 and duplicate genes were excluded. Screening the gene list from DEGs of three neuron subclusters NBNs, MGNs, and SPNs in the motor region at GW14 required pct.1 > 0.22, pct.2 < 0.8, and duplicate genes were excluded. Screening the gene list from DEGs of three neuron subclusters NBNs, MGNs, and SPNs in the somatosensory region at GW14 required pct.1 > 0.27 and duplicate genes were excluded. Screening the gene list from DEGs of three neuron subclusters NBNs, MGNs, and SPNs in the V1 region at GW14 required pct.1 > 0.13, pct.2 < 0.78, and duplicate genes were excluded. Screening the gene list from DEGs of NBNs, MGNs, and CPNs in neurons in the PFC region at GW18 required pct.1 > 0.16, pct.2 < 0.7, and duplicate genes were excluded. Screening the gene list from DEGs of NBNs, MGNs, and CPNs in neurons in the motor region at GW18 required pct.1 > 0.2, pct.2 < 0.9, and duplicate genes were excluded. Screening the gene list from DEGs of NBNs, MGNs, and CPNs in neurons in the somatosensory region at GW18 required pct.1 > 0.3, pct.2 < 0.9, and duplicate genes were excluded. Screening the gene list from DEGs of NBNs, MGNs, and CPNs in neurons in the parietal region at GW18 required pct.1 > 0.27 and duplicate genes were excluded. Screening the gene list from DEGs of NBNs, MGNs, and CPNs in neurons in the V1 region at GW18 required to exclude duplicate genes. Screening the gene list from DEGs of SP1, SP2, and SP3 from PFC, motor, somatosensory, parietal, and V1 regions in SPNs at GW18 required pct.1 > 0.21, pct.2 < 0.8, and duplicate genes were excluded. Screening the gene list from DEGs of NBNs, MGNs and CPNs in neurons in the parietal region at GW20 required pct.1 > 0.15 and duplicate genes were excluded. Screening the gene list from DEGs of SPNs and CPNs from PFC, parietal, temporal and V1 regions at GW20 required only to exclude duplicate genes.

### Pseudotime Expression Heatmap of 14 SPN Genes in the Lineage Tree at GW10

113 out of 124 SPN genes were detected in the RGCs, IPCs, and neurons at GW10. Due to the low proportion of IPCs, RGCs and IPCs cells were merged to calculate SPN‐specific gene expression levels. The overall expression levels of 113 SPN genes in RGCs and IPCs and their expression levels in neurons were calculated by “AverageExpression” function (default parameters), separately. Based on the overall expression levels of these SPN genes in RGCs and IPCs, 7 SPN genes that were highly expressed in RGCs and IPCs and had higher expression levels than neurons were selected (Table S11, Supporting Information). Similarly, 7 SPN genes were selected that were more highly expressed in neurons (Table S12, Supporting Information). The “pheatmap” function was used to map the pseudotime expression heatmap of these 14 SPN‐specific genes in the pseudotime developmental lineage tree constructed by RGCs, IPCs, and neurons at GW10.

### Expression Heatmaps of 10 Human‐Specific Genes

68 genes specifically present in humans were listed in relevant literature,^[^ ^48^ ^]^ of which 11 genes had been reported multiple times in existing studies.^[^ ^49^, ^50^, ^51^ ^]^ Cells at GW20 were more mature in development and differentiation, with a more diverse range of cell types, including SPNs and CPNs. Among these 11 genes, 8 genes were detected in the single‐cell RNA‐seq datasets at GW20. Based on the overall expression levels of these 8 genes calculated using “AverageExpression” function (default parameters) in NPCs and neurons, 5 genes with higher overall expression levels were selected (expression levels > 0.05). Out of the remaining 57 genes, 32 genes were detected at GW20. Using the “AverageExpression” function (default parameters) to calculate the overall expression levels of these 32 genes in NPCs and neurons and obtained the top 9 genes with higher overall expression levels (expression levels > 0.15). From these 14 human‐specific genes, 10 human‐specific genes were extracted with changed expression clusters, and using the “pheatmap” function (default parameters) to generate gene expression heatmaps for these 10 genes across various NPCs and neuron subclusters from GW10 to GW20.

### GO Analysis

GO enrichment analysis was performed using the R package “clusterProfiler” to explore function discrepancy.^[^ ^69^ ^]^ Gene‐GO terms network plot was created using the “cnetplot” function, and biological processes (BP), cellular components (CC) and molecular functions (MF) of genes were displayed by “barplot” function.

### Expression Heatmaps of 16 ECM Genes

108 ECM genes were confirmed in the existing literature.^[^ ^36^ ^]^ Cells at GW20 were more mature in development and differentiation, with a more diverse range of cell types, including SPNs and CPNs. The expression of these 108 ECM genes in single‐cell RNA‐seq datasets at GW20 was examined, of which 103 ECM genes were detected. The expression levels of these 103 ECM genes were calculated separately in NPCs and neurons (“AverageExpression” function), and the differential expression levels in these two cell clusters were also computed. By subtracting the expression levels of genes in neurons from those in NPCs, top 6 ECM genes with significant differences in NPCs were selected. Similarly, by subtracting the expression levels of genes in NPCs from those in neurons, top 10 ECM genes with significant differences in neurons were selected. Using the “pheatmap” function (default parameters), gene expression heatmaps were generated for these 16 genes across various NPCs and neuron subclusters from GW10 to GW20.

### Statistical Analysis

Datasets of spatial transcriptomics and single‐cell RNA‐seq were collected from brain samples at 8 different human fetal stages. The impact of the cell cycle on cell clustering and dimensionality reduction was eliminated using the “CellCycleScoring” function, and all data were normalized using the “NormalizeData” function and “LogNormalize” method. Gene markers for cell clusters were selected from genes of differential expressions with statistical significance (adjusted *P* < 0.05) using the “FindAllMarkers” function. In the GO enrichment analysis, GO terms of genes with statistical significance (*P* < 0.05) were selected. In the Pearson correlation analyses between different cell clusters based on spatial transcriptome datasets, labeled cell clusters with correlation (correlation coefficient ≥ 0.7, *P* < 0.05) were calculated using the “rcorr” function in the RStudio software.

## Conflict of Interest

The authors declare no conflict of interest.

## Author Contributions

X.Y.G. and T.S. conceived and designed the study. X.Y.G. performed the spatial transcriptomics and single‐cell RNA sequencing experiments. X.Y.G., T.L., J.S., and J.S. analyzed sequencing data. W.J.C. and Q.W.Y. prepared brain tissues. X.Y.G. and T.S. wrote the paper. T.S. supervised the entire study.

## Supporting information



Supporting Information

Supplemental Tables

## Data Availability

The data that support the findings of this study are openly available in Gene Expression Omnibus at https://www.ncbi.nlm.nih.gov/geo/query/acc.cgi?acc=GSE262082, reference number 262082.
