# Supplementary material for: Molecular Lineages and Spatial Distributions of Subplate Neurons in the Human Fetal Cerebral Cortex
Source: Adv Sci (Weinh). 2024 Nov 4;11(47):2407137. doi: 10.1002/advs.202407137 (PMC11653714; doi:10.1002/advs.202407137)
Supplement: Supplementary file 1 — Supporting Information [file ADVS-11-2407137-s002.pdf]

ADVANCED SCIENCE
Open Access

Supporting Information

for *Adv. Sci.*, DOI 10.1002/advs.202407137

Molecular Lineages and Spatial Distributions of Subplate Neurons in the Human Fetal Cerebral Cortex

*Xueyu Guo, Trevor Lee, Jason Sun, Julianne Sun, Wenjie Cai, Qingwei Yang and Tao Sun**

Supplementary figures

Molecular lineages and spatial distribution of subplate neurons in the human fetal cerebral cortex

Xueyu Guo[1], Trevor Lee[2], Jason Sun[3], Julianne Sun[3], Wenjie Cai[4], Qingwei Yang[5] and Tao Sun[1,6,*]

[1]Center for Precision Medicine, Huaqiao University, Xiamen, Fujian 361021, China

[2]Department of Cell and Developmental Biology, Weill Medical College of Cornell University, 1300 York Avenue, New York, NY 10065, USA

[3]Xiamen Institute of Technology Attached School, Xiamen, Fujian, China

[4]Department of Radiation Oncology, First Hospital of Quanzhou, Fujian Medical University, Quanzhou, Fujian, China

[5]Department of Neurology, Zhongshan Hospital, School of Medicine, Xiamen University, Xiamen, Fujian, China

[6]School of Medicine and School of Biomedical Sciences, Huaqiao University, Xiamen, Fujian, China.

[*]**Corresponding author**: Dr. Tao Sun, E-mail: taosun@hqu.edu.cn

Running title: Molecular landscape of human fetal subplate neurons.

Key words: Human fetal brain; cerebral cortex; subplate neurons; spatial transcriptomics; single-cell RNA-sequencing.

Figure S1

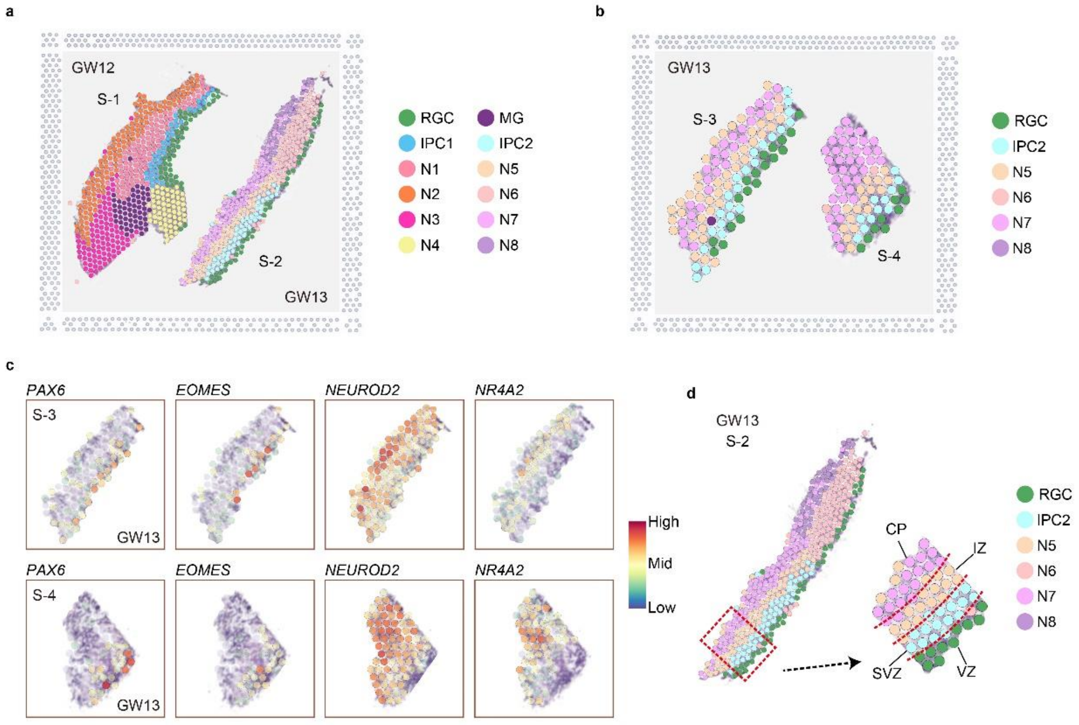

Figure S1. Spatial atlas of the human fetal cortices. a, Spatial distribution of cells in S-1 and S-2 under spatial spots clustering. Each spot represents one area in the sections. b, Spatial distribution of cells in S-3 and S-4 under spatial spots clustering. They are similar to those in S-2. c, Expression patterns of *PAX6*, *EOMES*, *NEUROD2*, and *NR4A2* in S-3 and S-4, respectively. d, Cell clusters and cell distribution based on spatial spots clustering in S-2. In the boxed area in S-2, RGC is in the VZ, IPC2 is in the SVZ, N5 is in the IZ and N7 is in the CP.

Figure S2

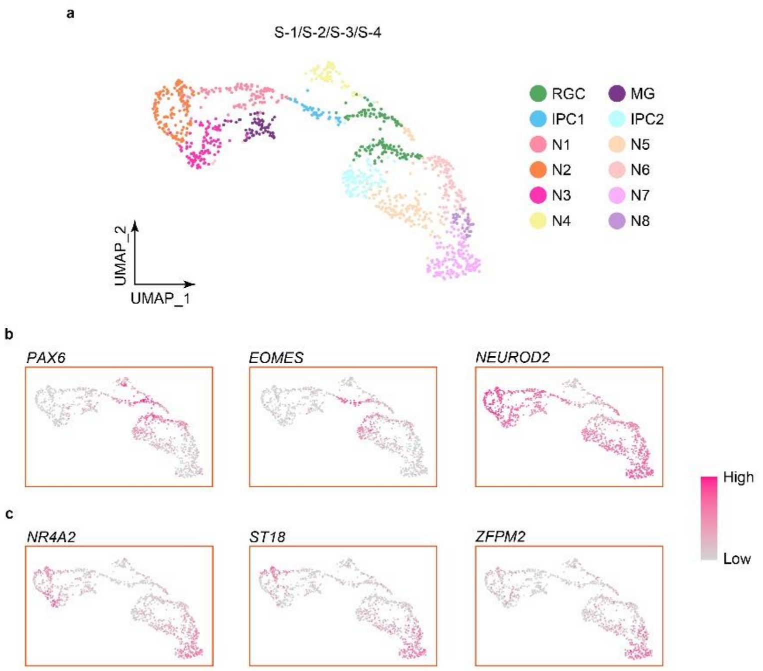

Figure S2. Expression patterns of canonical genes in S-1 to S-4 sections. a, Cell clusters in human fetal cortex sections (S-1, S-2, S-3 and S-4) visualized using UMAP after spatial spots clustering. Each dot represents one area in the sections. b, Expression patterns of *PAX6*, *EOMES* and *NEUROD2* at GW12 and GW13 visualized by the UMAP. c, Expression patterns of SPN markers *NR4A2*, *ST18* and *ZFPM2* at GW12 and GW13 visualized by the UMAP.

Figure S3

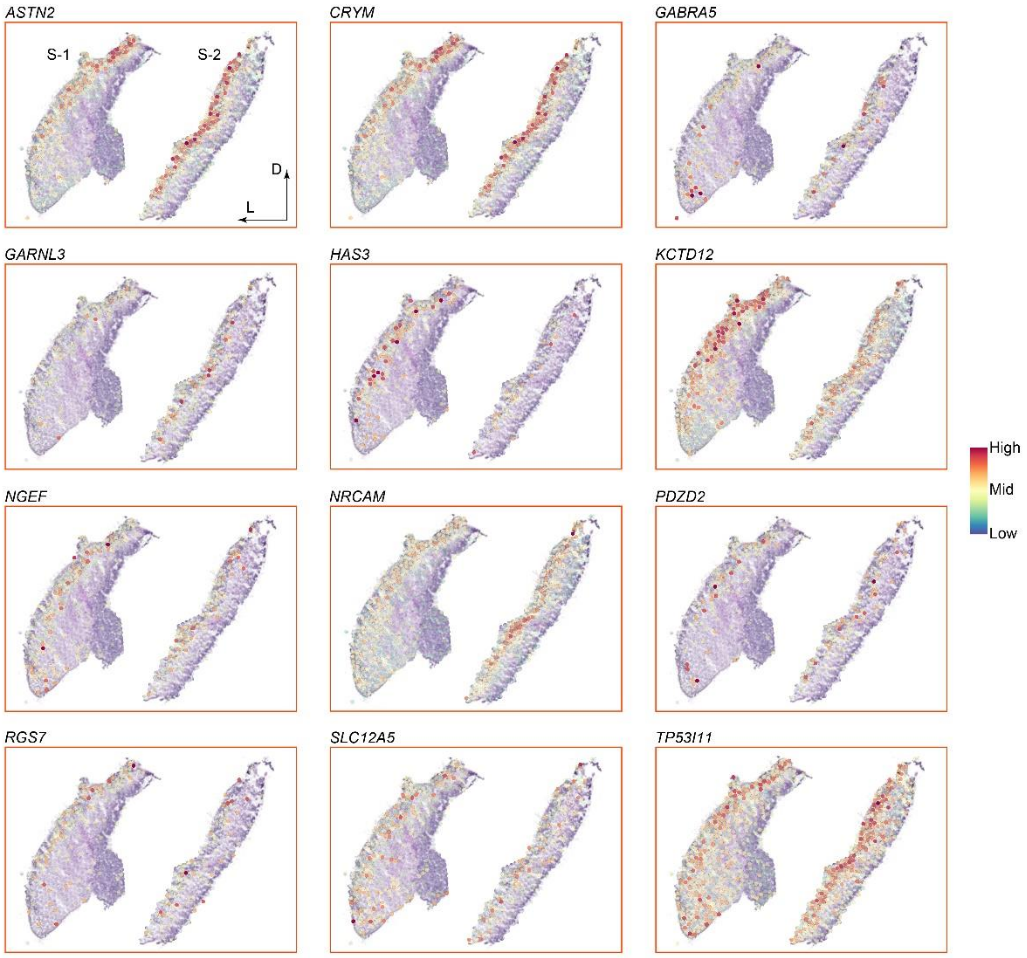

Figure S3. Expression patterns of SPN-specific genes in S-1 and S-2 at GW12 and GW13.

Figure S4

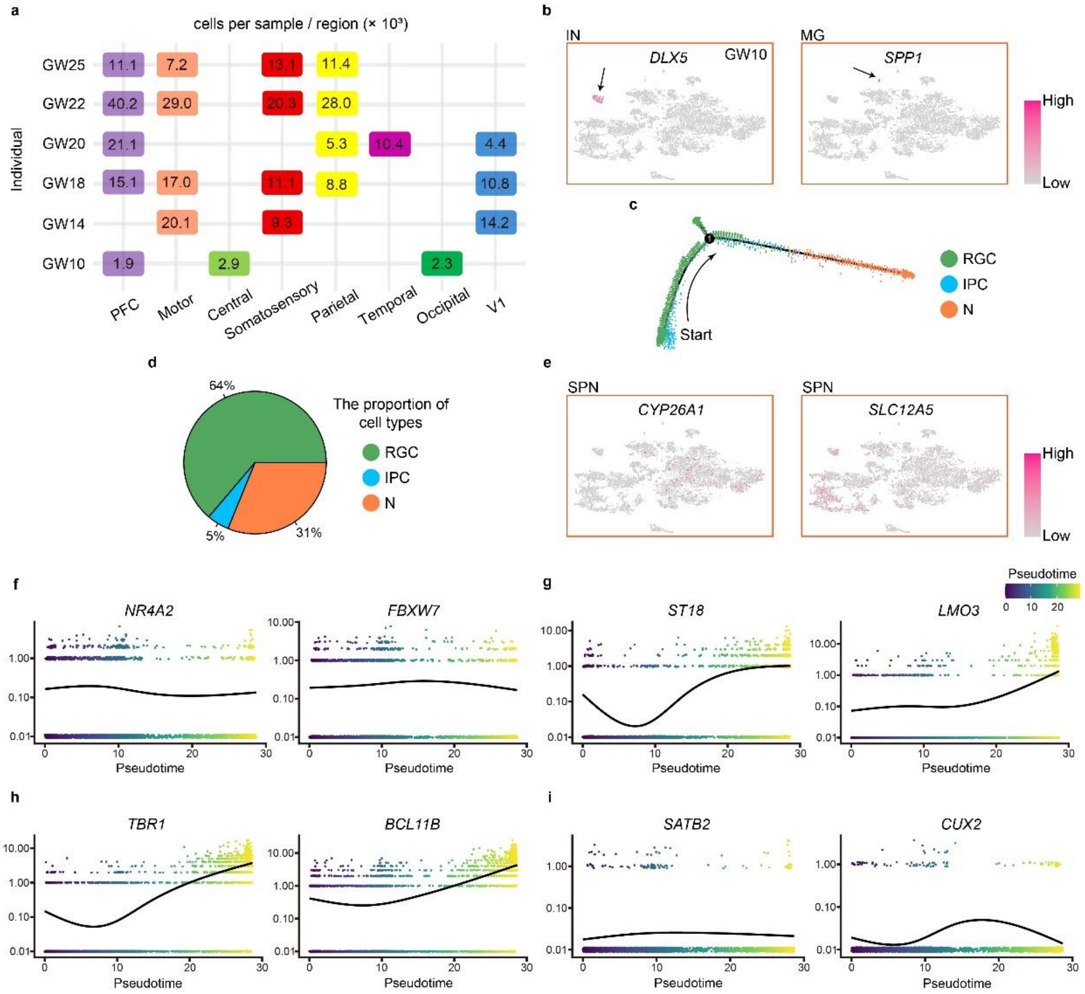

Figure S4. The differential expression of SPN-specific genes in the human fetal cortices at GW10. a, Matrix showing the distribution of the number of cells in different regions (PFC, motor, central, somatosensory, parietal, temporal, occipital and V1) for each of the individual samples (GW10, GW14, GW18, GW20, GW22 and GW25) of the selected single-cell RNA-seq datasets. The number of cells is shown in boxes, times 1000. Each color represents a region. b, Feature genes of INs and MG at GW10 visualized by t-SNE. Each dot represents one cell. c, The developmental trajectory of

RGCs, IPCs and neurons from PFC, central and occipital regions at GW10 constructed by Monocle 2. The black arrow indicates the direction of cell development and differentiation. d, Proportion of RGCs, IPCs and Ns from PFC, central and occipital regions in the number of cells at GW10. e, SPN-specific genes at GW10 visualized by t-SNE. f-i, Changes in the expression levels of SPN-specific genes highly expressed in RGCs (f), SPN-specific genes highly expressed in neurons (g), early-born neuron feature genes (h) and late-born neuron feature genes (i) along with pseudotime in the lineage tree (Figure 3e) at GW10.

Figure S5

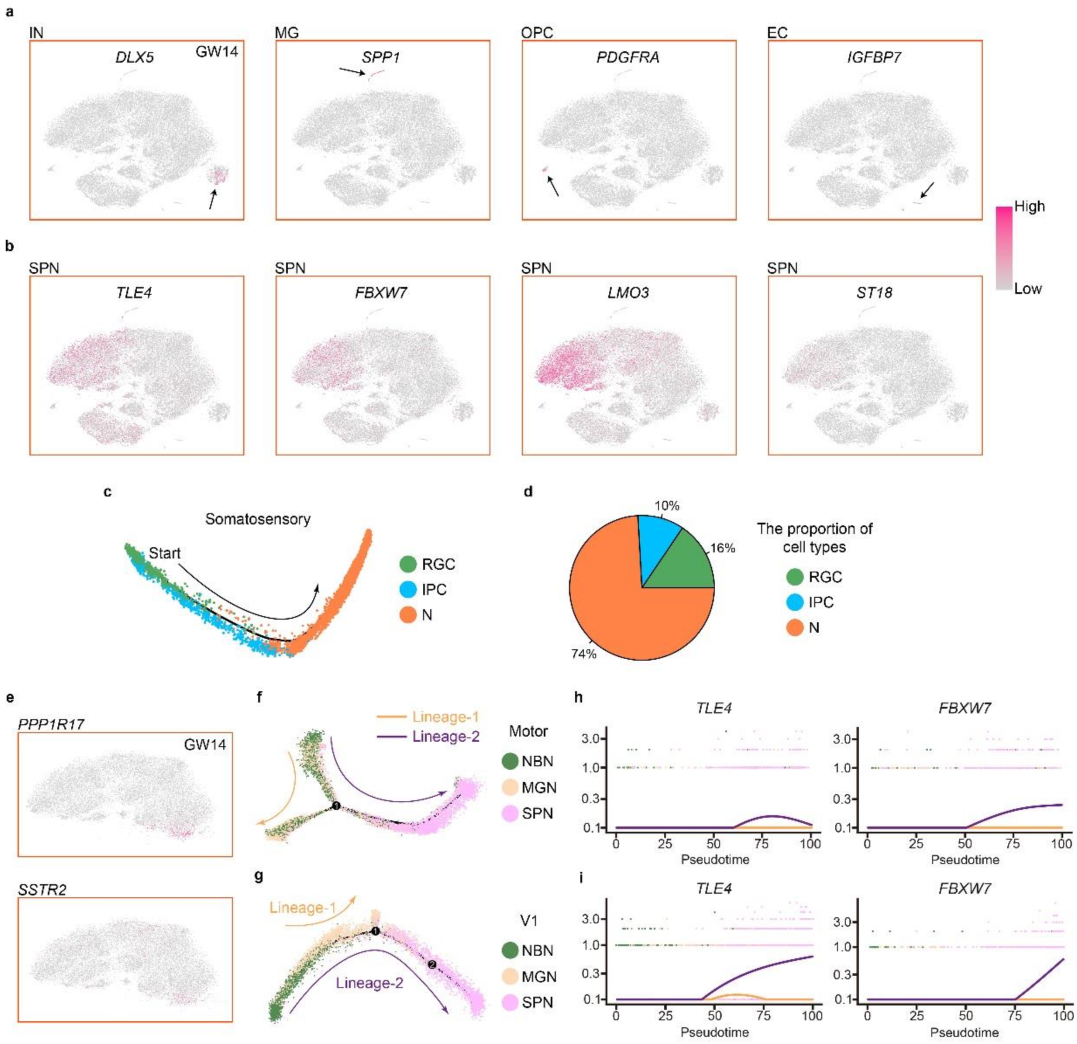

Figure S5. The neurogenesis in human fetal cortical subplate at GW14. a, Feature genes of INs, MG, OPCs and ECs at GW14 visualized by t-SNE. Each dot represents one cell. b, SPN-specific genes at GW14 visualized by t-SNE. c, The developmental trajectory of RGCs, IPCs and Ns from the somatosensory region constructed by Monocle 2. The black arrow indicates the direction of cell development and differentiation. d, Proportion of RGCs, IPCs and neurons from motor, somatosensory and V1 regions in the number of cells at GW14. e, Expression of marker genes of IPCs in neurons at

GW14 visualized by t-SNE. f, g, The developmental trajectories of neuron subclusters NBNs, MGNs and SPNs in the motor (f) and V1 (g) regions at GW14 constructed by Monocle 2, respectively. Colored arrows represent constructed Lineage-1 and Lineage-2. h, i, Changes in the expression levels of *TLE4* and *FBXW7* along pseudotime in different lineages in the developmental trajectories in the motor (h) and V1 (i) regions at GW14.

Figure S6

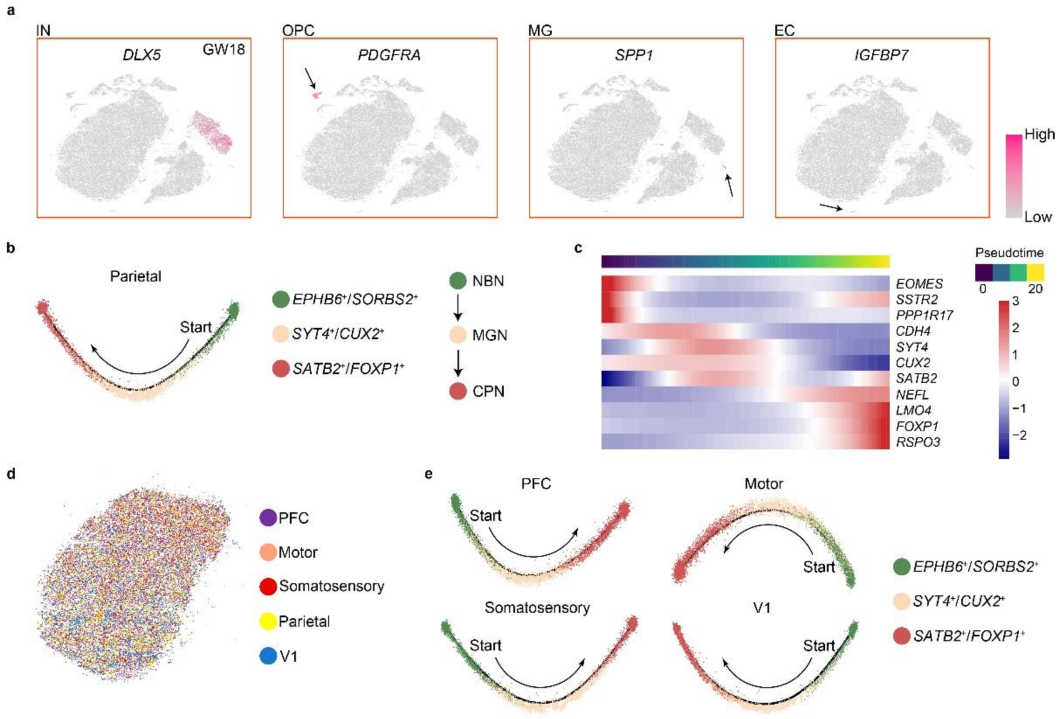

Figure S6. Development and differentiation of the non-SPN neuron subclusters at GW18. a, Feature genes of INs, OPCs, MG and ECs at GW18 visualized by t-SNE. Each dot represents one cell. b, The developmental trajectory of the non-SPN neuron subclusters NBNs, MGNs and CPNs from the parietal region at GW18 constructed by Monocle 2. The black arrow indicates the direction of cell development and differentiation. c, Pseudotime expression heatmap of high expression genes of NBNs, MGNs and CPNs in the developmental trajectory (b) at GW18. d, Clustering of the non-SPN neuron subclusters from PFC, motor, somatosensory, parietal and V1 regions visualized by t-SNE after batch correction. e, The developmental trajectories of the non-SPN neuron subclusters NBNs, MGNs and CPNs from the PFC, motor, somatosensory and V1 regions at GW18 constructed by Monocle 2.

Figure S7

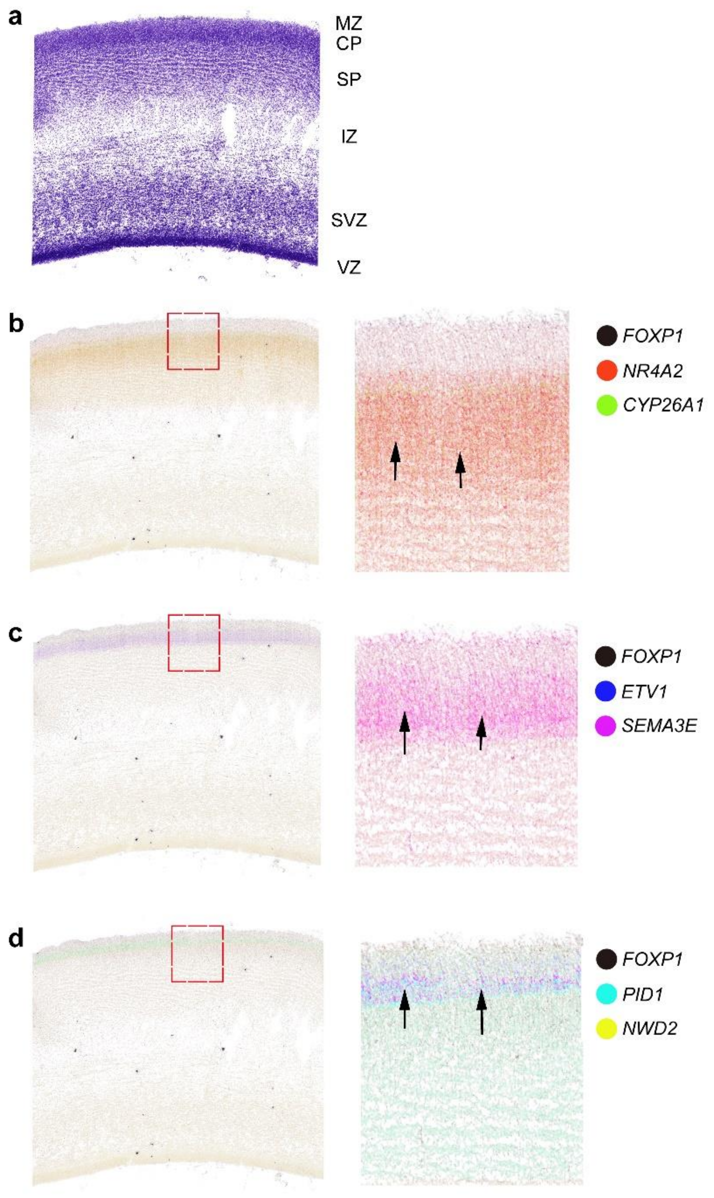

Figure S7. Expression patterns of genes specific for subplate neurons in sections of human fetal brains at GW17-18. a, Nissl staining on a reference section. MZ, marginal zone; CP, cortical plate; SP, subplate; IZ, intermediate zone; SVZ, subventricular zone; VZ, ventricular zone. b-c, Images of *in situ* hybridization generated in Allen Brain Atlas for *NR4A2*$^{+}$/*CYP26A1*$^{+}$ (SP1), *ETV1*$^{+}$/*SEMA3E*$^{+}$ (SP2) and *PID1*$^{+}$/*NWD2*$^{+}$ (SP3) were

merged in one section using pseudo colors. Boxed areas are shown in high power views with the highlight of gene expression patterns (arrows).

Figure S8

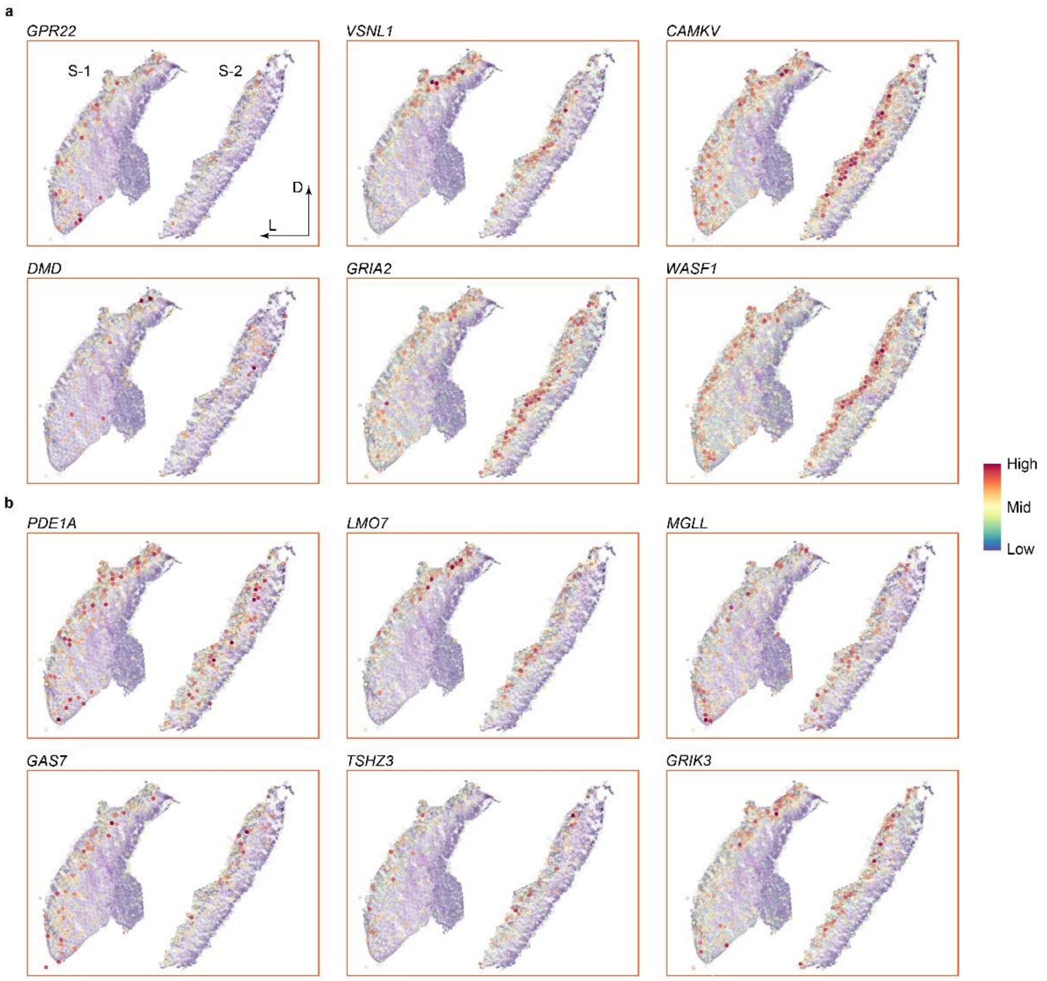

Figure S8. Expression patterns of new SPN-specific genes in S-1 and S-2. a, b, Spatial expression in S-1 and S-2 of new SPN-specific genes discovered in SPNs in the single-cell RNA-seq datasets at GW14 (a) and GW18 (b). Each spot represents one area in the sections.

Figure S9

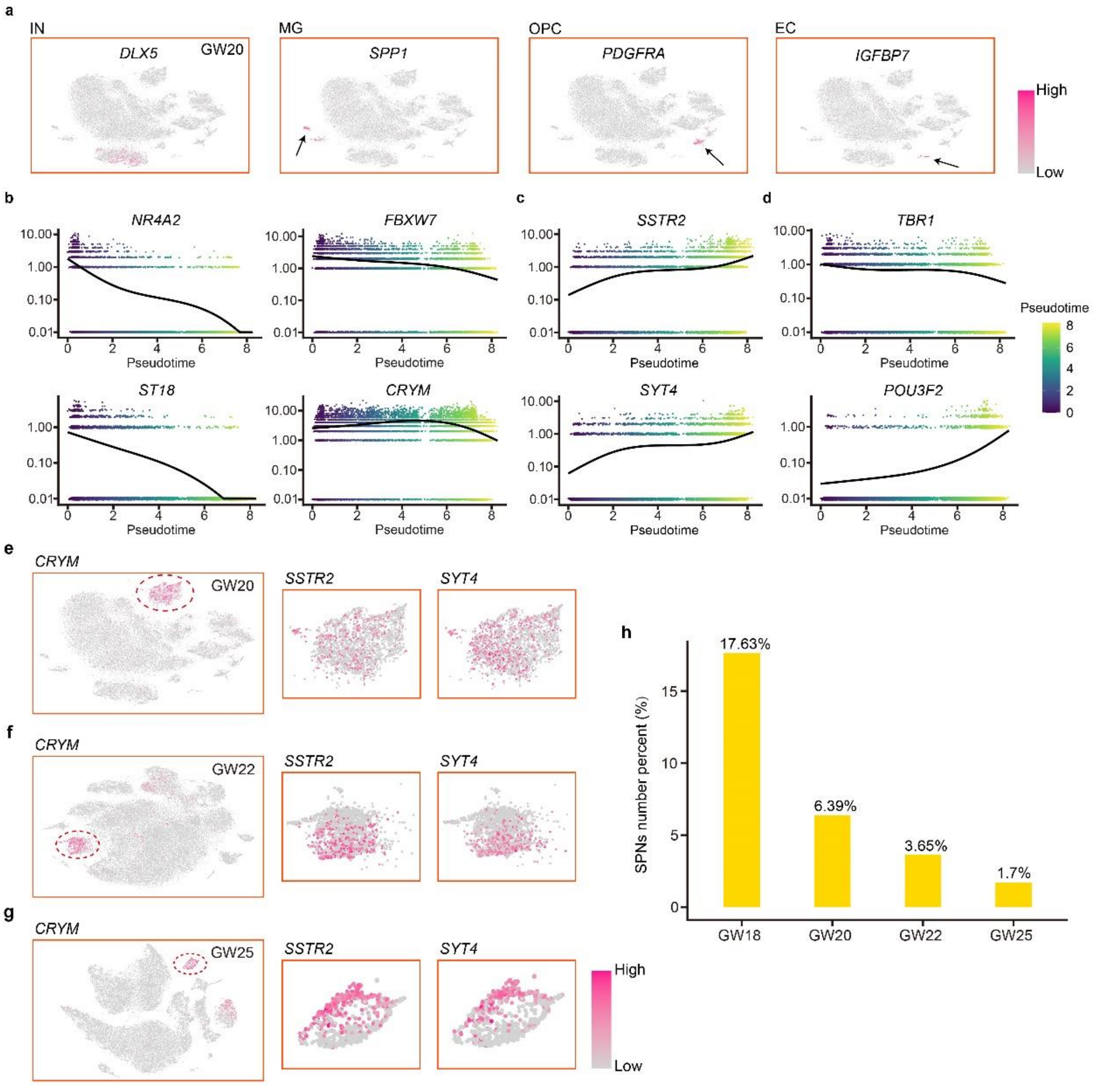

Figure S9. Characteristic changes in the differentiation process of subplate neurons. a, feature genes of INs, MG, OPCs and ECs at GW20 visualized by t-SNE. Each dot represents one cell. b-d, Changes in the expression levels of SPN-specific genes (b), IPC feature gene *SSTR2* and MGN feature gene *SYT4* (c), early-born neuron feature gene *TBR1* and late-born neuron feature gene *POU3F2* (d) along the pseudotime in the lineage tree at GW18 (Figure 4f). e-g, Localization of SPNs based on the expression of

SPN-specific gene *CRYM* at GW20 (e), GW22 (f) and GW25 (g), and visualizing the expression of IPC feature gene *SSTR2* and MGN feature gene *SYT4* in SPNs at each stage. h, The proportion of SPNs in the number of cells at different stages from GW18 to GW25.

Figure S10

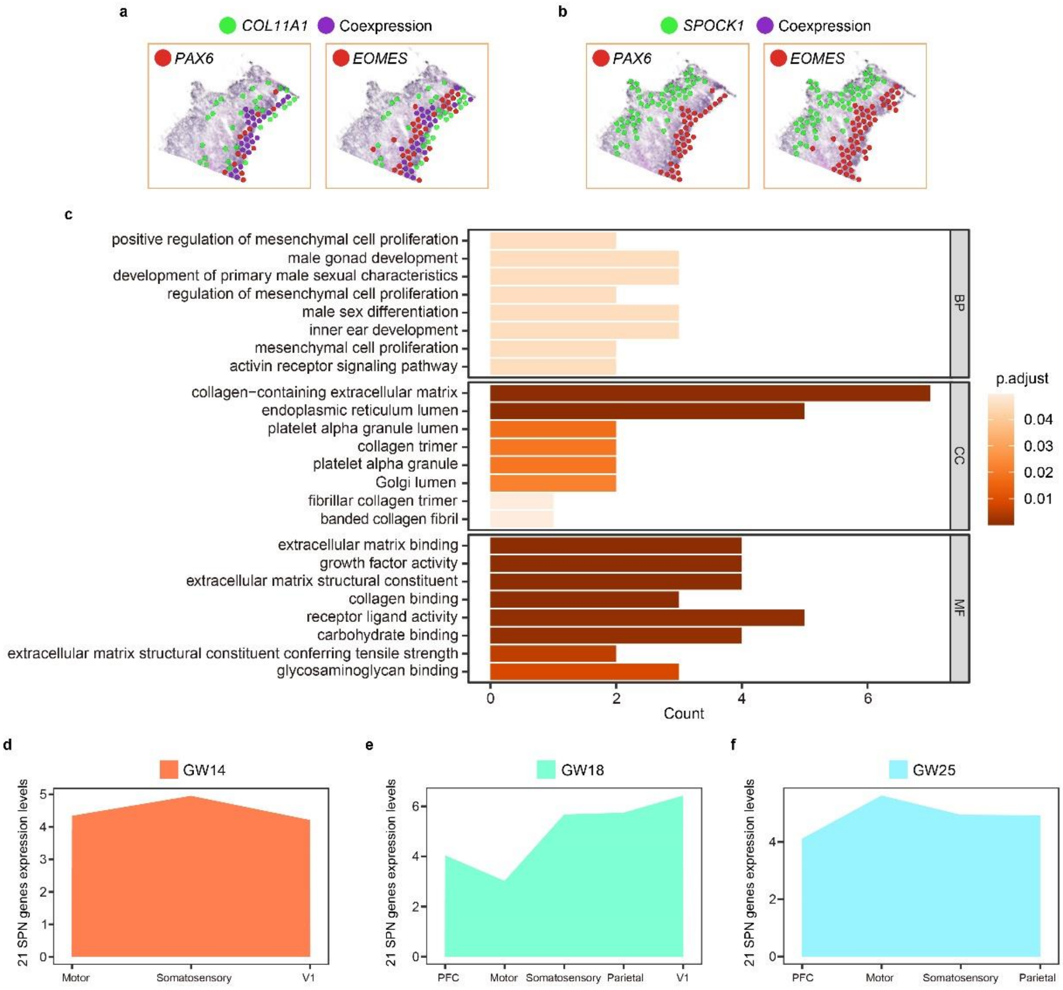

Figure S10. Analysis of ECM genes in the human fetal cortex. a, b, Spatial co-expression of the ECM gene *COL11A1* and *SPOCK1* with RGC feature gene *PAX6* and IPC feature gene *EOMES* in the high-power views of the boxed area in S-1 (Figure 6b), respectively. Each spot represents one area in the section. c, GO enrichment analysis describing representative 16 ECM genes from three aspects: biological processes (BP), cellular components (CC) and molecular functions (MF). Adjusted $P < 0.05$. d-f, Regional area plots of the sum of the expression levels of 21 ECM genes stably

expressed in NPCs in each region at GW14 (d), GW18 (e) and GW25 (f). Different colors represent different periods.
